# Broad and Efficient Activation of Memory CD4^+^ T Cells by Novel HAdV- and HCMV-Derived Peptide Pools

**DOI:** 10.3389/fimmu.2021.700438

**Published:** 2021-07-07

**Authors:** Alexander Höttler, Léo März, Maren Lübke, Hans-Georg Rammensee, Stefan Stevanović

**Affiliations:** ^1^ Department of Immunology, Institute for Cell Biology, University of Tübingen, Tübingen, Germany; ^2^ German Cancer Consortium (DKTK) and German Cancer Research Center (DKFZ), Partner Site Tübingen, Tübingen, Germany; ^3^ Cluster of Excellence iFIT (EXC2180) ‘Image-Guided and Functionally Instructed Tumor Therapies’, University of Tübingen, Tübingen, Germany; ^4^ German Center for Infection Research (DZIF), Partner Site Tübingen, Tübingen, Germany

**Keywords:** promiscuous CD4^+^ T-cell epitopes, epitope prediction, antiviral T cells, HCMV (human cytomegalovirus), HAdV (human adenovirus), peptide pools, adoptive T-Cell therapy, HLA class II

## Abstract

Reactivation of Human Cytomegalovirus (HCMV) and Human Adenovirus (HAdV) in immunocompromised patients following stem cell transplantation (SCT) or solid organ transplantation (SOT) is associated with high morbidity and mortality. The adoptive transfer of virus-specific CD8^+^ and CD4^+^ T cells has been shown to re-establish the antiviral T-cell response and improve clinical outcome. The viral load in immunocompromised patients can efficiently be reduced solely by the infusion of virus-specific CD4^+^ T cells. The identification of CD4^+^ T-cell epitopes has mainly focused on a limited number of viral proteins that were characterized as immunodominant. Here, we used *in silico* prediction to determine promiscuous CD4^+^ T-cell epitopes from the entire proteomes of HCMV and HAdV. Immunogenicity testing with enzyme-linked immuno spot (ELISpot) assays and intracellular cytokine staining (ICS) revealed numerous novel CD4^+^ T-cell epitopes derived from a broad spectrum of viral antigens. We identified 17 novel HCMV-derived and seven novel HAdV-derived CD4^+^ T-cell epitopes that were recognized by > 50% of the assessed peripheral blood mononuclear cell (PBMC) samples. The newly identified epitopes were pooled with previously published, retested epitopes to stimulate virus-specific memory T cells in PBMCs from numerous randomly selected blood donors. Our peptide pools induced strong IFNγ secretion in 46 out of 48 (HCMV) and 31 out of 31 (HAdV) PBMC cultures. In conclusion, we applied an efficient method to screen large viral proteomes for promiscuous CD4^+^ T-cell epitopes to improve the detection and isolation of virus-specific T cells in a clinical setting.

## Introduction

The period with most complications following stem cell transplantation (SCT) is the time of aplasia between the depletion of the host’s immune system and immune reconstitution. Opportunistic viral infections or reactivation of endogenous virus are a common threat during this period. The therapeutic options are limited and associated with severe side-effects of antiviral agents and emerging resistance (1). Frequent complications following SCT are caused by Epstein-Barr virus, Human Cytomegalovirus (HCMV), and Human Adenovirus (HAdV) infections (2). These viruses are known to persist in the human organism after primary infection and can be reactivated during aplasia. HCMV and HAdV are highly prevalent: approximately 60% (3) of the population have encountered HCMV. A study by Sukdolak et al. identified HAdV-specific antibodies in 196 of 204 healthy individuals (96%) (4). These viral infections are also a threat to other patients with acquired immunodeficiency, like solid organ transplant (SOT) recipients and HIV-infected individuals (5).

Viral replication is effectively controlled by virus-specific CD4^+^ and CD8^+^ T cells (6–9). Hence, the adoptive T-cell transfer was shown to be a promising treatment approach for refractory viral infections (10–16). The identification of frequently recognized epitopes is a prerequisite for the isolation of virus-specific T cells for adoptive transfer (13). For this purpose, *in silico* epitope prediction followed by immunogenicity testing is a well-established method (17, 18).

The screening for HCMV and HAdV epitopes has mainly been restricted to a limited number of viral proteins that were characterized as immunodominant (19–21). Other studies showed that the antiviral T-cell response is directed against a broad spectrum of antigens (17, 22). Numerous CD8^+^ T-cell epitopes from various antigens have been discovered ever since. The vital role of CD4^+^ T cells for viral clearance has been emphasized (11, 12, 23, 24). The repertoire of frequently recognized CD4^+^ T-cell epitopes is nevertheless limited and the source antigen spectrum has been barely characterized.

The promiscuous binding of CD4^+^ T-cell epitopes to different HLA class II allotypes is well-known (25). However, there is no established approach for their systematic identification. Promiscuous epitopes bear the advantage of being recognized by a wider range of individuals of a population. *In silico* prediction of CD4^+^ T cell epitopes is still challenging due to the binding of peptides of variable length by HLA class II molecules and impeded attribution of HLA class II peptide-binding motifs to certain allotypes (26). As a consequence, the experimentally known HLA class II ligands can only be found among the best 10-15% of the predicted HLA class II epitopes compared to HLA class I prediction methods where the ligands are ranked within the top 1-2% (27).

Here we used SYFPEITHI (28) to predict frequently recognized CD4^+^ T-cell epitopes from the reviewed proteomes of HCMV and HAdV (29). By performing enzyme-linked immunospot (ELISpot) assays with peripheral blood mononuclear cells (PBMCs), we were able to identify 17 novel immunodominant (recognized by > 50% of the assessed PBMC samples) HCMV- and seven novel immunodominant HAdV-derived T-cell epitopes. The immunodominant epitopes stimulated multifunctional CD4^+^ T cells in intracellular cytokine staining (ICS). Further, we were able to design one peptide pool containing 14 HCMV-derived CD4^+^ T-cell epitopes and one peptide pool containing 12 HAdV-derived epitopes that were able to induce an IFNγ secretion in 46 out of 48 (95.8%) and 31 out of 31 (100.0%) randomly selected PBMC samples, respectively. The newly-identified epitopes are derived from a broad spectrum of viral source proteins of all temporal classes of viral protein expression and various functions.

## Material and Methods

### Prediction and Selection of Promiscuous CD4^+^ T-Cell Epitopes

**Figure 1:**
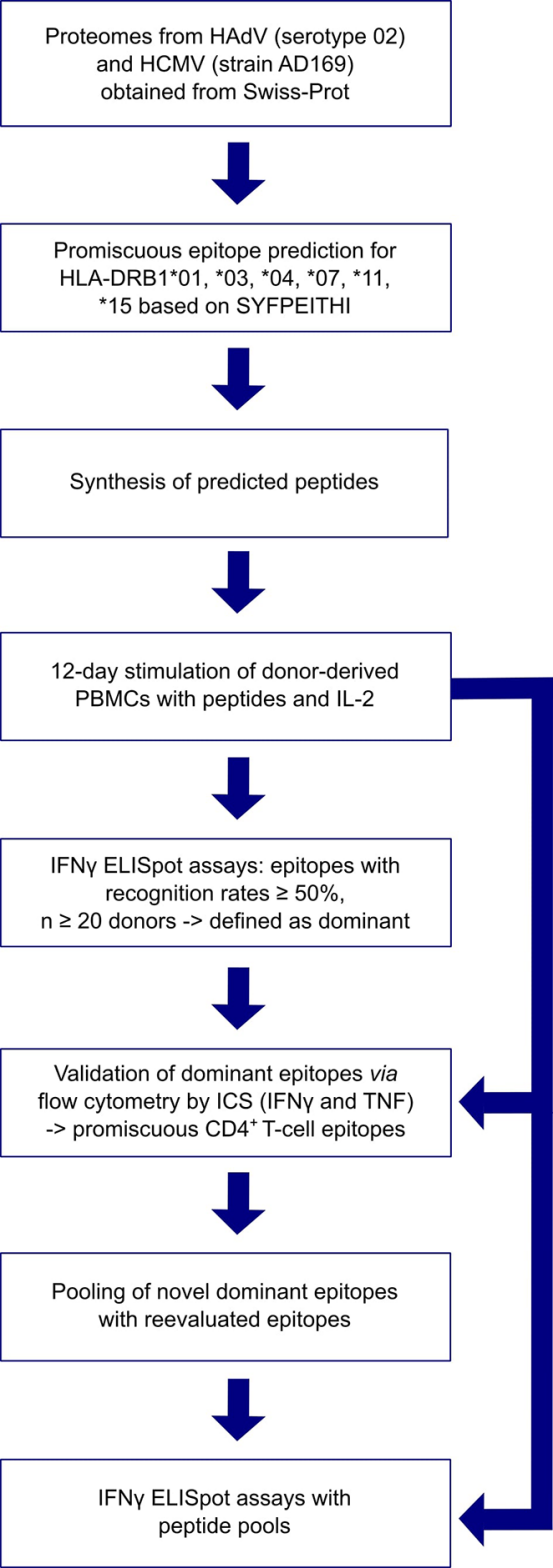
Overview of applied methods.

**Figure 2:**
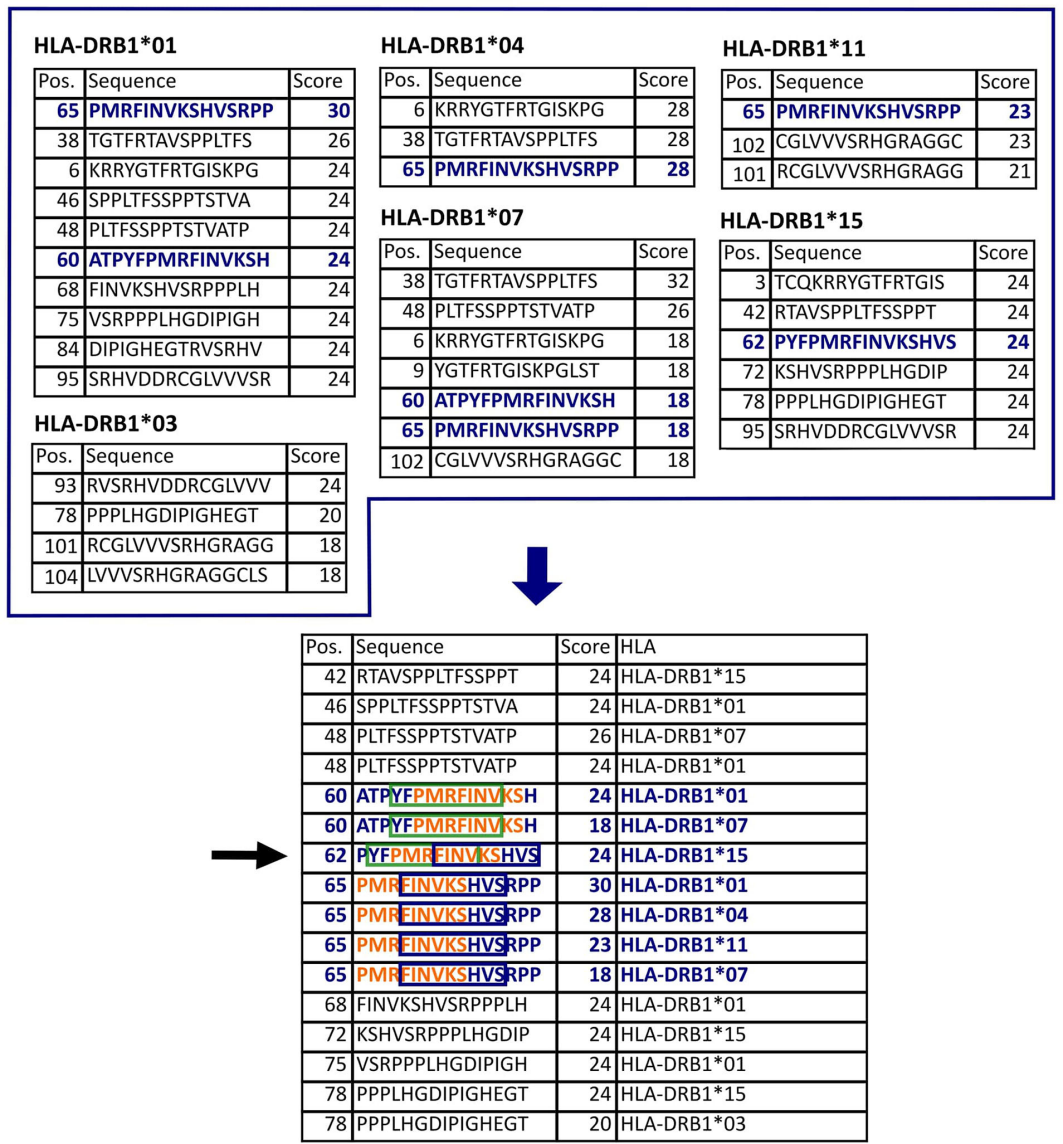
Epitope selection example for protein US4. The upper tables list the 2% highest scored peptides for each of the HLA allotypes of interest. This protein is 119 aa long. Thus, there are 105 possible 15-mers. In this case, the 2% highest scores equal to 2.1, so the three highest scored peptides were selected (all peptides with the smallest selected score value were considered, see HLA-DRB1*01,03,07,15). The lower table lists all selected peptides sorted by their position in the protein. The nine-aa-long binding-core (orange letters, green box, and blue box) is essential for the stable binding of a peptide to an HLA molecule. The binding core of the selected epitope candidate could be shifted three positions either to the N- or to the C-terminus. Accordingly, the peptide 62-76 (black arrow) potentially binds to five different HLA allotypes (in blue) since it contains the binding cores of seven peptides that are predicted binders to these HLA allotypes.

**Figure 1** provides an overview of the methods applied in this project. The proteomes of HCMV (strain AD169) and HAdV (serotype 02, species C) were screened for peptides that potentially bind to several of the six HLA class II allotypes available for prediction with SYFPEITHI (28). These cover the most frequent HLA class II allotypes in the German population (HLA-DRB1*01, HLA-DRB1*03, HLA-DRB1*04, HLA-DRB1*07, HLA-DRB1*11, HLA-DRB1*15) (allelefrequencies.net, Germany pop 8), except for HLA-DRB1*13 (30). Reviewed source protein sequences were obtained from the Swiss-Prot section of the UniProt Knowledgebase (29). Epitopes were predicted for each protein separately. **Figure 2** depicts the selection process of epitope candidates for synthesis and testing. In brief, the 2% highest scored epitope candidates for each of the HLA allotypes of interest were determined. Subsequently, these epitope candidates were screened for nine amino-acid-long binding-core sequences that appeared multiple times. The binding core is the anchoring region of the epitope and is defined as the central nine amino acid (aa) sequence of the predicted 15-mer epitope candidate that is flanked by three aa residues on the N- and C-terminal ends. The binding core sequences of the different epitope candidates should be shifted by three aa at maximum to the N- or C-terminus in relation to the later synthesized peptide. In this way, the tested peptide contains the binding cores of several epitope candidates for different HLA allotypes.

The further epitope selection process differed slightly between HAdV and HCMV epitope candidates due to the differing genome size.

#### HCMV

An HCMV epitope candidate was considered a promiscuous binder if it contained binding cores of epitope candidates predicted for at least five different HLA-DR allotypes. This way, we selected 169 peptides (**Table S2**). We initially tested the immunogenicity of several of these peptides in ELISpot assays and determined the recognition rates. Throughout the project, it turned out that epitope candidates that reached the maximum prediction score (top score) for one of the HLA-DR allotypes of interest were more frequently recognized by PBMC cultures in ELISpot assays. Hence, testing was narrowed to top scorers and epitopes predicted to be binders to all six HLA allotypes (**Table S2**). Ten epitope candidates could not be synthesized in a satisfying quality. In total, 103 HCMV-derived peptides were synthesized and tested in ELISpot assays.

#### HAdV

An HAdV epitope candidate was considered a promiscuous binder if it contained at least five other core sequences of epitopes predicted for any of the six HLA-DR allotypes. The promiscuous epitope candidates that contained five and four cores were additionally predicted for the same six HLA class II allotypes *via* NetMHCIIpan 2.0 (31). Epitopes that were predicted to be strong binders to at least five different HLA allotypes were synthesized and screened for immunogenicity. Based on the *in silico* prediction with SYFPEITHI, 27 epitope candidates met the selection criterion to contain at least six cores of the top-2% predicted peptides. Three of the predicted epitopes could not be synthesized in a satisfying purity. Furthermore, three epitope candidates met the criterion to contain at least four core sequences and to be predicted as a strong binder to at least five of the screened HLA-DRB molecules by NetMHCIIpan 2.0. **Table 1** lists all tested HAdV-derived epitope candidates that emerged from the promiscuous SYFPEITHI prediction. In total, 27 predicted peptides were synthesized and further investigated.

**Table 1:** ELISpot and ICS results of HAdV epitope candidates.

n_cores_	Protein_Position_	Sequence	ELISpot		ICS	Reference
			n_pd_/n_td_	rr		
**4**	PKG1_292-306_*	VSKFFHAFPSKLHDK	16/20	80.0	CD4	this paper
**7**	E3145_46-60_*	RGIFCVVKQAKLTYE	18/24	75.0	CD4	this paper
**7**	**COR10_53-67_**	LPLLIPLIAAAIGAV	17/24	70.8	CD8	this paper
**6**	LEAD_32-46_*	TLVLAFVKTCAVLAA	17/24	70.8	CD4	this paper
**4**	E1BS_130-144_*	KNRLLLLSSVRPAII	14/20	70.0	CD4	this paper
**6**	CAP3_28-42_*	RQVMDRIMSLTARNP	16/24	66.7	CD4	this paper
**4**	E3GL_140-154_*	TLLYLKYKSRRSFID	13/20	65.0	CD4	this paper
**8**	CAPSH_65-79_*	TLRFIPVDREDTAYS	13/21	61.9	CD4	(18)
**6**	COR10_64-78_*	IGAVPGIASVALQAQ	13/24	54.2	CD4/CD8	this paper
**6**	**PKG1_353-367_**	LNRFVNTYTKGLPLA	11/24	45.8	CD4	this paper
**6**	**Y215_75-89_**	TPKLILSNSLSGSSS	10/23	43.5	nt	this paper
**7**	**UXP_48-62_**	KDLLTDFKAFAARFS	6/16	37.5	nt	this paper
**6**	**DPOL_491-505_**	YLKVMVRDTFALTHT	4/16	25.0	nt	this paper
**7**	**E3GL_5-19_**	ILGLLALAAVCSAAK	3/13	23.1	nt	this paper
**6**	**E4RF2_67-81_**	MRVIISVGSFVMVPG	3/16	18.8	nt	this paper
**7**	**E3145_77-91_**	QKLVLMVGEKPITVT	2/13	15.4	nt	this paper
**7**	**E1A_20-34_**	LDQLIEEVLADNLPP	2/13	15.4	nt	this paper
**7**	**PKG3_242-256_**	LLDLINILQSIVVQE	2/15	13.3	nt	this paper
**6**	**PKG2_70-84_**	LATVPSIATTSAPQA	2/15	13.3	nt	this paper
**6**	**SF33K_70-84_**	LATVPSIATTSAPQA	2/15	13.3	nt	this paper
**9**	**E1A_243-257_**	IHPVVPLCPIKPVAV	1/8	12.5	nt	this paper
**6**	**CORE5_72-86_**	VRRVLRPGTTVVFTP	1/8	12.5	nt	this paper
**8**	**E4RF1_79-93_**	YIMTPDMTEELSVVL	0/8	0.0	nt	this paper
**7**	**Y172_31-45_**	ISPFIKLTSTHSANK	0/8	0.0	nt	this paper
**7**	**Y137_48-62_**	CAVVDALDRAKGEPV	0/8	0.0	nt	this paper
**6**	**SHUT_386-400_**	LVSYLGILHENRLGQ	0/8	0.0	nt	this paper
**6**	**Y215_149-163_**	VTAFRCIIQGHPRGP	0/15	0.0	nt	this paper
**6**	**E434_142-156_**	ASWFRMVVDGAMFNQ	0/8	0.0	nt	this paper
**7**	**E4RF4_18-32_**	CVGWLGVAYSAVVDV	X	X	nt	this paper
**6**	**LEAD_76-90_**	VWLVVFYFGCLSLTV	X	X	nt	this paper
**6**	**E3RDB_6-20_**	IFVLLIFCALPVLCS	X	X	nt	this paper

In total, 31 epitope candidates were identified by our prediction. The aa sequences of SF33K_70-84_ and PKG2_70-84_ are identical. The left column shows the number of contained top-2% cores. All epitopes that were predicted to contain at least six top-2% cores were synthesized. The epitopes that were predicted to contain four to five cores were screened for potential HLA class II binding by NetMHCIIpan 2.0. The epitopes that were predicted as strong binders to at least five of the HLA class II allotypes were also synthesized. Only the epitope CAPSH_65-79_ was previously published. n_cores_ , number of best 2% cores; n_pd_ , number of positively tested PBMC cultures; n_td_ , number of tested PBMC cultures; rr, ELISpot recognition rate; X, peptide could not be synthesized to a satisfying purity; nt, not tested. An asterisk indicates that the peptide was contained in the pool.

### Peptide Synthesis and Composition of Peptide Pools

Selected peptides were synthesized by standard 9-fluorenylmethyloxycarbonyl/tert-butyl strategy using Activo P11 (Activotec) or Liberty Blue (CEM). Peptide purity was analyzed by reversed-phase HPLC (e2695, Waters) and identity confirmed by nano-UHPLC (UltiMate 3000 RSLCnano) coupled online to a hybrid mass spectrometer (LTQ Orbitrap XL, both Thermo Fisher Scientific).

Peptides were lyophilized and dissolved in 20 mg/ml DMSO. Depending on solubility most peptides were diluted 1:10 in bidistilled H_2_O and stored at -80°C.

#### HCMV

For the HCMV peptide pool, 14 peptides at a concentration of 10 mg/ml were pooled. The pp65^-^ HCMV peptide pool contains all peptides of the HCMV peptide pool, except for pp65-derived peptides. Peptides were centrifuged, and the supernatant was used if they could not be dissolved entirely.

#### HAdV

The HAdV peptide pool comprises 12 peptides.

### Cell Culture

PBMCs were extracted from buffy coats by Ficoll-Hypaque density gradient centrifugation. Buffy coats were kindly provided by the Institute for Clinical and Experimental Transfusion Medicine at the University Hospital of Tübingen. Written informed consent has been obtained consistent with the Declaration of Helsinki and applicable laws and regulations, which has been approved by the Ethik-Kommission an der Medizinischen Fakultät der Eberhard-Karls-Universität am Universitätsklinikum Tübingen (Project No. 507/2017B01). HCMV serostatus and two-digit HLA-A and -B typing were provided by the Institute for Clinical and Experimental Transfusion Medicine. HAdV serology testing and HLA class II typing were not performed. Four-digit numbers were assigned to the PBMC samples in our laboratory. PBMCs were randomly selected for ELISpot screening assays. Cells were stored at -80°C in FCS and 10% DMSO. Cells were thawed, transferred to well-plates, and incubated (7.5% CO_2_ and 37°C in humidified incubators) for one day before further treatment. The culture medium consisted of IMDM (Thermo Fisher Scientific) supplemented with 5% heat-inactivated pooled human plasma (isolated from healthy blood donors), 100 U/ml penicillin, 100 µg/ml streptomycin (Sigma-Aldrich), 25 µg/ml gentamicin (Life Technologies), and 50 µM β-mercaptoethanol (Carl Roth).

### IFNγ ELISpot Assay

In a standard screening ELISpot duplicates of donor-derived PBMC cultures were stimulated with nine different peptides. The peptide FLNA_HUMAN_1169-1683_ or DMSO served as negative controls. Single peptides or peptide pools (**Table S1**) and phytohemagglutinin (PHA; Sigma Life Science) were used as positive controls. The assay was conducted after a 12-day stimulation with the epitope candidates and interleukin-2 (IL-2). The epitope candidates and the negative control peptides were added on day 1 (24 h after thawing) at a final concentration of 5 µg/ml per peptide. IL-2 was added on days 2, 5, and 7 at a final concentration of 20 U/ml. Nitrocellulose plates (Immunospot M200, Merck Millipore) were coated with anti-human IFNγ (1 mg/ml mAb1-D1K, Mabtech AB) at a final concentration of 2 µg/ml one day before seeding of the harvested PBMCs (500,000 cells/well). Peptides were added to the wells at a final concentration of 2.5 µg/ml per peptide. An ELISpot kit (Mabtech) was used to detect the IFNγ release of stimulated cells following the manufacturer’s recommendation. In brief, the well membranes were coated with a primary IFNγ capture antibody before peptides and cells were added. After 20 to 22h of incubation a biotinylated secondary antibody and an avidin-alkaline phosphatase conjugate were added to the wells. Sigmafast BCIP/NBT tablets were used to obtain purple spots indicating an activated cell. Automatic spot counting was implemented by the ImmunoSpot S5 analyzer (CTL) and ImmunoSpot software (v5.1). A T-cell reaction to a peptide of interest was evaluated as positive when the mean spot count exceeded ten and was threefold higher than the negative control. PBMC cultures that showed high background responses in the negative control were excluded from the analysis.

#### HCMV

The PBMC samples that were screened for immune responses against predicted HCMV epitopes were obtained from HCMV-seropositive blood donors. Spot counts were cut off at 2,000 per 1x10^6^ PBMCs.

#### HAdV

The PBMC samples that were screened for immune responses against HAdV-derived epitope candidates were obtained from blood donors without HAdV serology testing. Spot counts were cut off at 2,000 per 1x10^6^ PBMCs.

### Intracellular Cytokine Staining

Dominant CD4^+^ T-cell epitopes (≥ 50% recognition rate in ELISpot, number of tested donors ≥ 20) were validated *via* flow cytometry. PBMCs underwent a 12-day stimulation with the peptides and IL-2 as described before (32). Subsequently, 10^6^ cells were incubated with the peptide of interest or negative control peptide (FLNA_HUMAN_1169-1683_, f.c. 10 µg/ml for HCMV and 25 µg/ml for HAdV) or phorbol myristate acetate (PMA) and ionomycin as the positive control. Next, Brefeldin A (Sigma-Aldrich) and GolgiStop (BD Biosciences) were added before a 12 h incubation. Afterwards, cells were washed and stained with aqua live/dead fluorescent reactive dye (Life Technologies). The surface molecules CD4 and CD8 were marked with anti-human CD4 APC-Cy7 (Biolegend) and anti-human CD8 PE-Cy7 (Beckman Coulter) for HCMV and anti-human CD8-PerCP (Biolegend) for HAdV, fixated and permeabilized (Cytofix/Cytoperm; BD Biosciences). Intracellular cytokines were stained using anti-human TNF Pacific Blue™ (Mab11, Biolegend) and anti-human IFNγ (B27, Biolegend). Between each step, the plates were kept for 20 min at 4°C. The samples were measured one day after staining with a FACSCanto II cytometer (BD Biosciences). Data analysis was performed with FlowJo v10. Results were evaluated as positive if the response to the peptides of interest was threefold higher than to the negative control peptide and ≥ 0.1%.

### Alignment

Sequence alignment of correspondent epitope source proteins of different virus strains was conducted with the Multiple Sequence Alignment tool Clustal Omega (33). Only strains with available reviewed source protein sequences were considered.

### Data analysis

Data were analyzed using GraphPad Prism (v6).

## Results

### Epitope Candidates Induce Strong IFNγ Responses in PBMCs of Randomly Selected Donors

**Figure 3:**
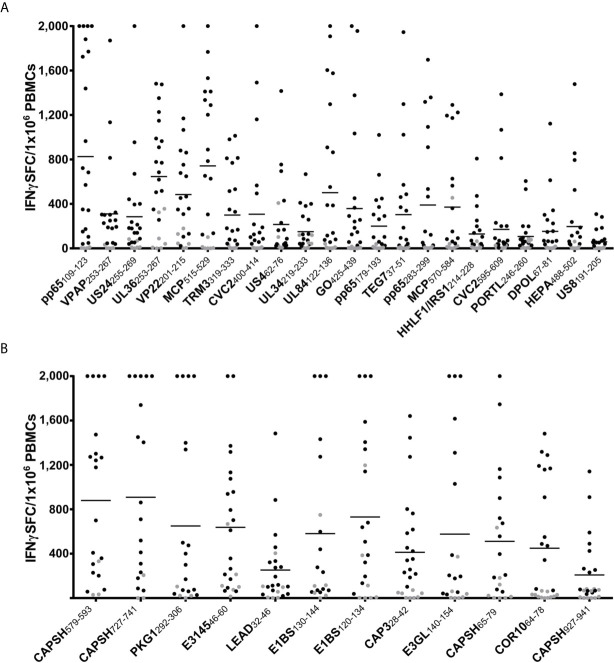
ELISpot screening results of promiscuous epitopes. IFNγ T-cell responses in ELISpot assays after 12-day stimulation with peptide and IL-2 to dominant HCMV **(A)** and HAdV **(B)** epitopes. The number of spot-forming cells (SFC) per 1x10^6^ PBMCs is plotted for each dominant epitope (negative control subtracted, cut-off at 2,000 SFC/1x10^6^). Each dot represents one tested PBMC culture (n > 20 per epitope). The T-cell response was defined as positive if the spot counts were threefold higher than in the negative control and higher than ten. Negative results are indicated in grey. The horizontal lines show the mean spot counts of all tested PBMC cultures for each peptide. The peptides are sorted according to their recognition rates from highest (left) to lowest (right).

The epitope candidates were tested in single-peptide ELISpot screening assays. The ELISpot assays were conducted after a 12-day stimulation with peptides and IL-2. The recognition rate is defined as the proportion of IFNγ-secreting PBMC cultures of all tested cultures. Epitopes with recognition rates of ≥ 50% are termed dominant epitopes, while epitopes with recognition rates of < 50% are defined as subdominant. The recognition rates of the epitope candidates are listed in **Tables 2** and **S3** (HCMV), and **Table 1** (HAdV). Response intensity (spot forming cells/1x10^6^ cells) varies between different donors and tends to increase with a higher recognition rate (**Figure 3**). We performed *ex vivo* ELISpots for several dominant epitopes. The recognition was generally low and below the positivity threshold for most epitopes.

**Table 2:** ELISpot and ICS results of HCMV epitope candidates.

Protein_position_	Sequence	ELISpot		ICS
		n_pd_/n_td_	rr	
**US24_255-269_**	SRRWWWAVRANLATP	17/23	73.9	CD4
**UL36_253-267_**	QYVLVDTFGVVYGYD	17/23	73.9	CD4
**UL24_201-215_**	KRYFRPLLRAWSLGL	16/22	72.7	CD4 & CD8
**MCP_515-529_**	DFVVTDFYKVGNITL	15/21	71.4	CD4
TRM3_319-333_*	FNTILGFLAQNTTKI	16/23	69.6	CD4
CVC2_400-414_*	RDDVLSLWSRRLLVG	14/22	63.6	CD4
**US4_62-76_**	PYFPMRFINVKSHVS	14/22	63.6	CD4
**UL34_219-233_**	NSFLHLLMNSGLDIA	14/22	63.6	CD4
UL84_122-136_*	RDPFQILLSTPLQLG	15/24	62.5	CD4
GO_425-439_*	LLFLDEIRNFSLRSP	15/24	62.5	CD4
**MCP_570-584_**	FHELRTWEIMEHMRL	12/20	60.0	CD4
HHLF1/IRS1_214-228_*	FRVFVYDLANNTLIL	13/22	59.1	CD4
CVC2_595-609_*	EHGLGRLLSVTLPRH	15/26	57.7	CD4
**PORTL_246-260_**	VRVFKKVRSERLEAQ	13/23	56.5	CD4
DPOL_67-81_*	LMFYREIKHLLSHDM	13/23	56.5	CD4
HEPA_488-502_*	FWQIQSLLGYISEHV	13/24	54.2	CD4
US8_191-205_*	MVLLLGYVLARTVYR	12/24	50.0	CD4

Epitopes with ELISpot recognition rates ≥ 50% are shown (tested epitope candidates with lower recognition rates: see **Table S3**). n_pd_ , number of positively tested PBMC cultures; n_td_ , number of tested PBMC cultures; rr, ELISpot recognition rate. An asterisk indicates that the peptide was contained in the pool.

#### HCMV

**Figure 4:**
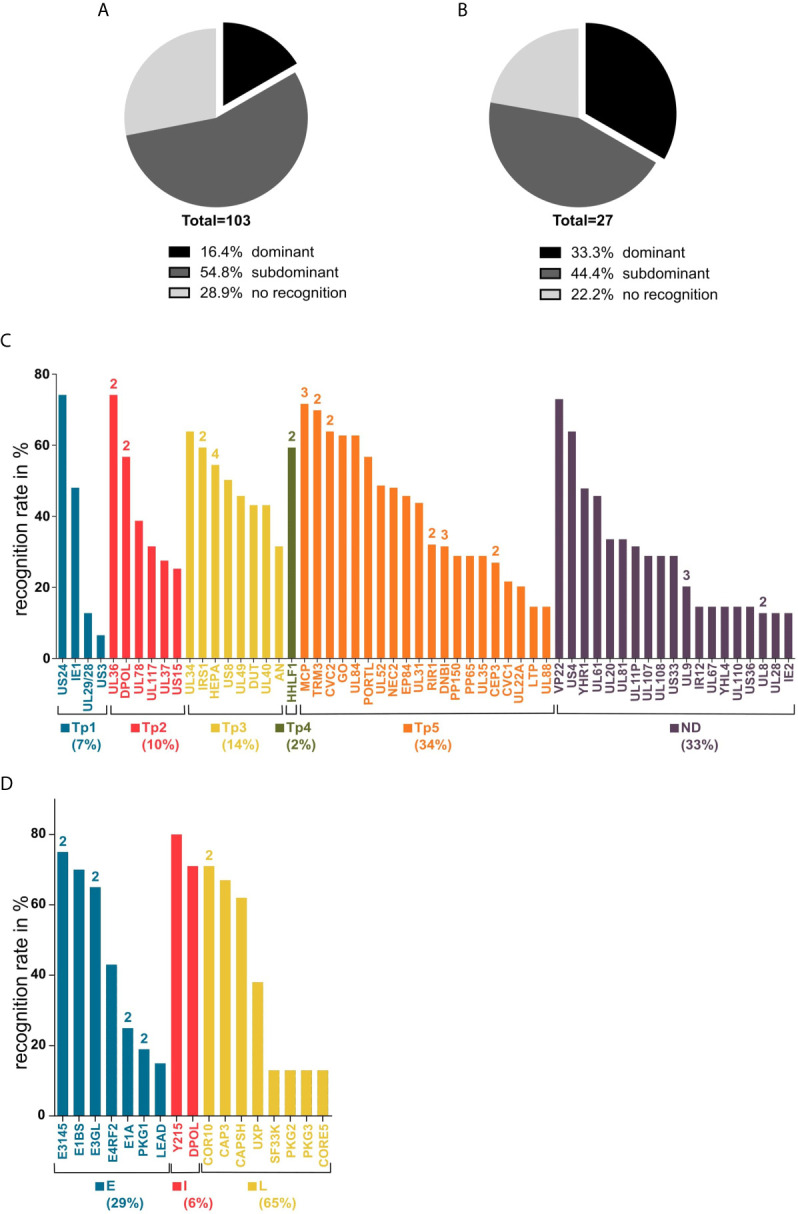
Recognition rates in ELISpot and source antigen spectrum. Panels **(A, B)** show the recognition rates (rr) of epitope candidates determined in ELISpot assays after a 12-day stimulation with peptides and IL-2. **(A)** all tested HCMV peptides, **(B)** all tested HAdV peptides. Dominant = rr ≥ 50%; subdominant = rr < 50%; no recognition = no T-cell response detected. Graphs **(C, D)** show the source antigen spectrum of dominant and subdominant epitopes. For each source protein, the epitope with the highest recognition rate is shown (ELISpot recognition rate in percent). The numbers above the bars indicate the number of epitopes from the respective antigen. **(C)** Tp1-5, temporal classes of protein expression as defined by Weekes et al. (34); ND, temporal class not determined. **(D)**. E, early; I, intermediate; L, late (29, 35–39).

Of the 103 predicted epitopes that were tested in ELISpot assays, 74 (71.2%) induced IFNγ release in at least one PBMC culture and are, therefore, T-cell epitopes. **Figure 4A** shows the proportion of dominant and subdominant epitopes of all assessed peptides. The 74 identified immunogenic epitopes originate from 58 different proteins. The 17 identified immunodominant epitopes are derived from 14 different source proteins. The spectrum of source proteins covers all temporal stages of protein expression, as defined by Weekes et al. (34). **Figure 4C** shows all source proteins and the ELISpot recognition rate of one representative epitope per protein. The majority of epitopes cluster in temporal class (tp) five (34%), followed by tp3 (14%), tp2 (10%), tp1 (7%), and tp4 (2%).

#### HAdV

The single-peptide ELISpot screening assays of 27 epitope candidates resulted in the identification of 21 HAdV-derived T-cell epitopes, nine classified as immunodominant and twelve as subdominant. One of the immunodominant epitopes, CAPSH_65-79_, was previously identified (18). From the 24 epitope candidates identified by the promiscuous SYFPEITHI prediction only considering epitopes that were predicted to contain at least six core sequences, six (25.0%) are dominant epitopes and twelve (50.0%) are subdominant epitopes (**Figure 4B**). All three epitopes (PKG1_292-306_, E1BS_130-144_, E3GL_140-154_) that were predicted by the combined SYFPEITHI-NetMHCIIpan 2.0 prediction were shown to be immunodominant. The 22 T-cell epitopes, including CAPSH_65-79_, originate from 17 adenoviral source proteins. The eight newly identified immunodominant epitopes are derived from seven different adenoviral source proteins, which are transcribed in early, intermediate, and late time points of viral replication (**Figure 4D**).

### Dominant T-cell Epitopes Trigger IFNγ and TNF Secretion Predominantly in CD4^+^ T Cells

**Figure 5:**
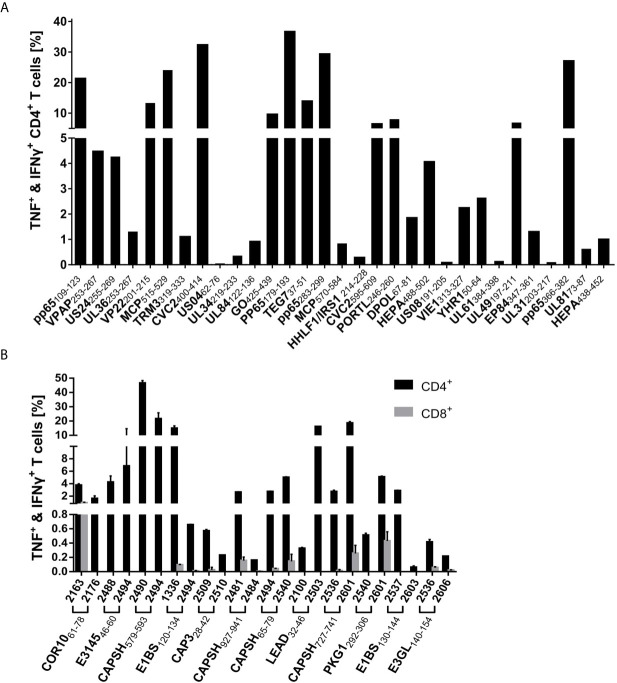
ICS characterization of virus-specific T cells. Dominant epitopes identified in ELISpot assays were validated in ICS after a 12-day stimulation with peptide and IL-2. IFNγ and TNF responses were evaluated as positive if threefold higher than in the negative control and ≥ 0,1% **(A)** Percentage of cytokine-positive CD4^+^ T cells of one representative PBMC culture per HCMV epitope. **(B)** Mean percentage of IFNγ^+^TNF^+^ CD4^+^ (black) and CD8^+^ (grey) T cells for all tested PBMC cultures (n = 2 per HAdV epitope).

**Figure 6:**
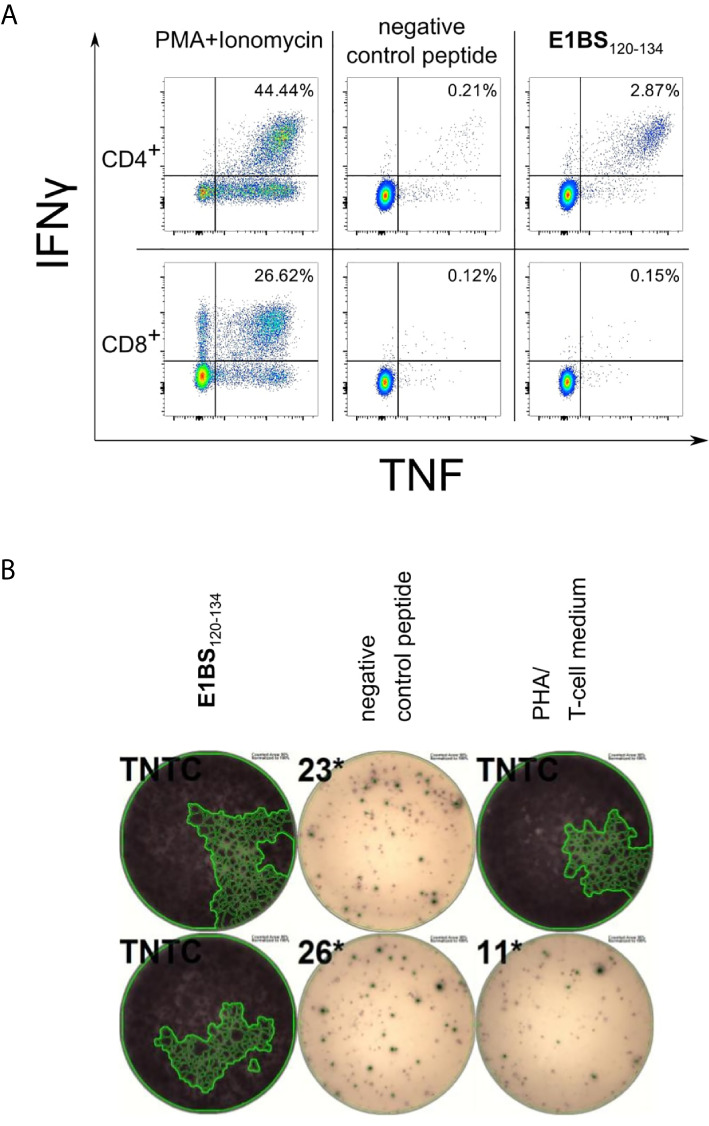
Representative results of ELISpot and ICS for E1BS_120-134_ using PBMCs of donor 2494. PBMCs of healthy blood donor 2494 were pre-stimulated with peptide and IL-2 for 12 days before ELISpot assay and ICS. PBMCs were gated on lymphocytes, single cells, viable cells, parallel on CD4 or CD8, and on IFNγ and TNF in ICS. **(A)** ICS results of the HAdV-derived epitope E1BS_120-134_. Upper plots are gated on CD4^+^ positive cells and lower plots on CD8^+^ cells. **(B)** ELISpot results of E1BS_120-134_ in donor 2494 and the negative control peptide in duplicates. PHA, positive control. TNTC, too numerous to count.

Recognition rates of dominant T-cell epitopes were assessed in ELISpot assays using PBMCs of 20 different donors at minimum. Successively, the epitope-specific T cells were validated and characterized *via* intracellular cytokine staining of TNF and IFNγ. Cytokine secretion by CD4^+^ T cells was confirmed for all dominant epitopes except for the HAdV epitope COR10_53-67_ (**Tables 1**–**4** and **Figures 5A, B**). As observed in ELISpot assays, the proportion of cytokine-secreting cells varied considerably between the different epitopes (**Figures 5A, B**). **Figure 6** shows a representative ICS result and the corresponding ELISpot assay of peptide E1BS_120-134_ with PBMCs of donor 2494.

**Table 3:** ELISpot and ICS results of re-evaluated published HCMV epitopes and epitopes from previous projects.

Protein_position_	Sequence	Elispot		ICS	Reference
		n_pd_/n_td_	rr		
PP65_109-123_*	MSIYVYALPLKMLNI	19/23	82.6	CD4 & CD8	(40)
VPAP_253-267_*	YVASRNGLFAVENFL	16/21	76.2	CD4	–
PP65_179-193_*	DVYYTSAFVFPTKDV	13/21	61.9	CD4	(41) (177-191); (42) (177-191); (40) (180-194)
TEG7_37-51_*	LQAFLDENFKQLEIT	14/23	60.9	CD4	–
PP65_283-299_*	KPGKISHIMLDVAFTSH	12/20	60.0	CD4	(40); (41) (285-299); (20) (285-299); (42) (281-295)

Previously published HCMV epitopes and epitopes from recent research projects in our laboratory were reevaluated in ELISpot assays and ICS after a 12-day stimulation with peptide and IL-2. Epitopes with ELISpot recognition rates ≥ 50 are shown. n_pd_ , number of positively tested PBMC cultures; n_td_ , number of tested PBMC cultures; rr, ELISpot recognition rate. An asterisk indicates that the peptide was contained in the pool.

**Table 4:** ELISpot and ICS results of published HAdV epitopes, epitopes that were identified in previous work *via* non-promiscuous HLA class II epitope prediction and CAPSH_727-741_.

Protein_Position_	Sequence	ELISpot		ICS	Reference
		n_pd_/n_td_	rr		
CAPSH_579-593_*	PQKFFAIKNLLLLPG	18/21	85.7	CD4	(18)
CAPSH_727-741_*	TFYLNHTFKKVAITF	17/20	85.0	CD4	–
E1BS_120-134_*	MHLWRAVVRHKNRLL	15/22	68.2	CD4	–
CAPSH_927-941_*	DEPTLLYVLFEVFDV	13/24	54.2	CD4	(43, 44)
**CAPSH_492-506_**	NIALYLPDKLKYNPT	10/21	47.6	nt	–
**CAPSH_592-606_**	PGSYTYEWNFRKDVN	5/11	45.5	CD4	(18)
**CAPSH_354-368_**	TGNMGVLAGQASQLN	2/5	40.0	nt	(18)
**E1BS_28-42_**	WRFLWGSSQAKLVCR	6/16	37.5	nt	–
**E1B55_217-231_**	RCSMINMWPGVLGMD	5/13	36.5	nt	–
**CAPSH_ADE05_118-132_**	GTAYNALAPKGAPNP	5/14	35.7	nt	(18)
**CAPSH_935-949_**	LFEVFDVVRVHQPHR	7/21	33.3	nt	–
**CAPSH_621-635_**	GASIKFDSICLYATF	3/13	23.1	nt	(18)
**CAPSH_ADE05_209-223_**	SQWYETEINHAAGRV	3/14	21.4	nt	(18)
**CAPSH_598-612_**	EWNFRKDVNMVLQSS	4/21	19.1	neg	(18)
**CAPSH_530-544_**	VDCYINLGARWSLDY	1/6	16.7	nt	(18)
**CAPSH_734-748_**	FKKVAITFDSSVSWP-NH2	1/6	16.7	nt	(18)
**CAPSH_471-485_**	GNNFAMEINLNANLW	1/6	16.7	nt	(18)
**CAPSH_633-647_**	ATFFPMAHNTASTLE	2/14	14.3	nt	(18)
**DPOL_1006-1020_**	LKSVYGDTDSLFVTE	2/14	14.3	nt	–
**CAPSH_ADE05_423-437_**	TETLTKVKPKTGQEN	1/8	12.5	nt	(18)
**DPOL_528-542_**	VNQFYMLGSYRSEAD	1/8	12.5	nt	–
**DPOL_982-996_**	RAFVSEWSEFLYEED	1/8	12.5	nt	–
**DPOL_803-817_**	FPEWRCVAREYVQLN	1/8	12.5	nt	–
**CAPSH_693-707_**	GWAFTRLKTKETPSL	1/10	10.0	nt	(18)

Results of the immunogenicity testing of already published adenoviral HLA class II epitopes derived from the hexon protein (CAPSH), epitopes that were identified via non-promiscuous prediction in previous work, and CAPSH_727-741_ that resulted from the elongation of a 10mer HLA class II epitope. Only immunogenic peptides are listed. The epitopes that are derived from HAdV5 are marked with _ADE05 behind the protein name. All other epitopes are derived from HAdV2. n_pd_ , number of positively tested PBMC cultures; n_td_ , number of tested PBMC cultures; rr, ELISpot recognition rate; nt, not tested; neg, negative ICS result. An asterisk indicates that the peptide was contained in the pool.

#### HCMV

VP22_201-215_ (predicted) and pp65_109-123_ [published (40)] were the only peptides that also stimulated CD8^+^ T cells.

#### HAdV

From the nine dominant epitopes identified *via* promiscuous prediction, epitope COR10_53-67_ was the only epitope to show a response of CD8^+^ T cells in both tested PBMC cultures in ICS (1.4% and 4.0% of IFNγ^+^TNF^+^ cells of the viable CD8^+^ T-cell fraction) with no detectable CD4^+^ T-cell response. The epitope COR10_64-78_ showed stimulation of CD4^+^ and CD8^+^ T-cells in PBMCs of donor 2163 with a predominant activation of the CD4^+^ T-cell population. Since exclusively CD4^+^ T-cell activation was observed in the PBMC sample 2176, it was categorized as a CD4^+^ T-cell epitope.

### Peptide Pools Activate the Vast Majority of Tested PBMC Cultures

**Figure 7:**
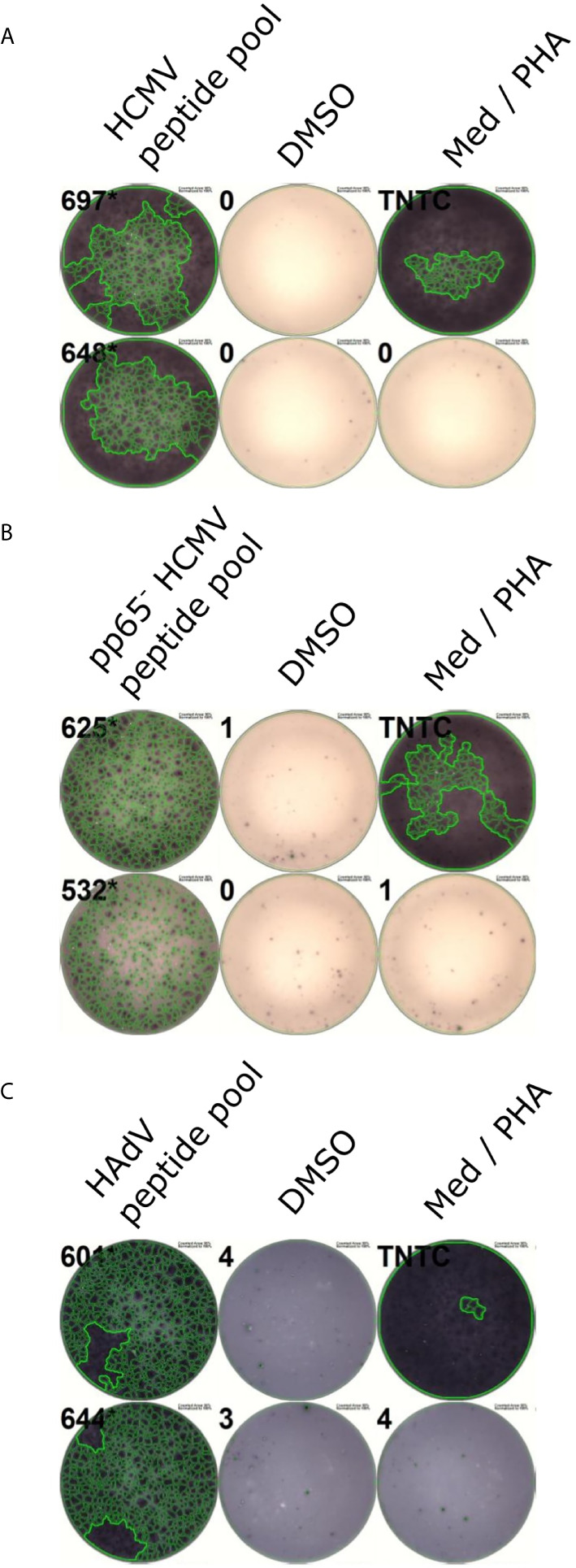
Representative ELISpots of peptide pools. PBMCs were incubated with peptide pools in ELISpot assays after a 12-day stimulation. Representative ELISpot wells for the HCMV peptide pool **(A)**, the pp65^-^ HCMV peptide pool **(B)**, and the HAdV peptide pool **(C)** are shown. DMSO served as a negative control, PHA as a positive control. Med, Medium control.

We pooled dominant CD4^+^ T-cell epitopes to create peptide pools that are recognized by virus-specific T cells of the majority of the population, regardless of their HLA alleles. To further increase the recognition rate of the pools, we included previously identified CD4^+^ T-cell epitopes. These epitopes were already published or discovered in previous projects in our laboratory and are listed in **Table 3** (HCMV) and **Table 4** (HAdV). They underwent the same immunogenicity testing procedure as the predicted epitopes. The ELISpot results of one representative donor per pool are shown in **Figure 7**.

#### HCMV

The peptide pool comprises 14 HCMV-derived epitopes (indicated by an asterisk in **Tables 2**, **3**), nine of which arose from the promiscuous prediction and were not published before (TRM3_319-333_, CVC2_400-414_, UL84_122-136_, GO_425-439_, HHLF1 & IRS1_214-228_, CVC2_595-609_, DPOL_67-81_, HEPA_488-502_, US8_191-205_).

The peptide pool activated T cells in 46 (95.8%) out of 48 randomly selected PBMC cultures from HCMV-positive donors (**Figure 7A**). To estimate the contribution of pp65 to the immunogenicity of the peptide pool, we tested the pool after excluding all pp65-derived epitopes. This modified peptide pool achieved a recognition rate of 30 out of 30 (**Figure 7B**).

#### HAdV

The HAdV-derived HLA class II peptide pool contains 12 epitopes (indicated by an asterisk in **Tables 1**, **4**) that were proven to be dominant CD4^+^ T-cell epitopes *via* ELISpot and ICS testing. Seven CD4^+^ T-cell epitopes (E3145_46-60_, CAP3_28-42_, COR10_64-78_, LEAD_32-46_, PKG1_292-306_, E1BS_130-144_, E3GL_140-154_) were newly identified *via* promiscuous epitope prediction.

The HAdV-derived HLA class II peptide pool was able to activate 31 out of 31 PBMC cultures from healthy blood donors (**Figure 7C**).

### The Peptide Sequences of Dominant Epitopes are Conserved Among HCMV Strains or HAdV Serotypes

**Figure 8:**
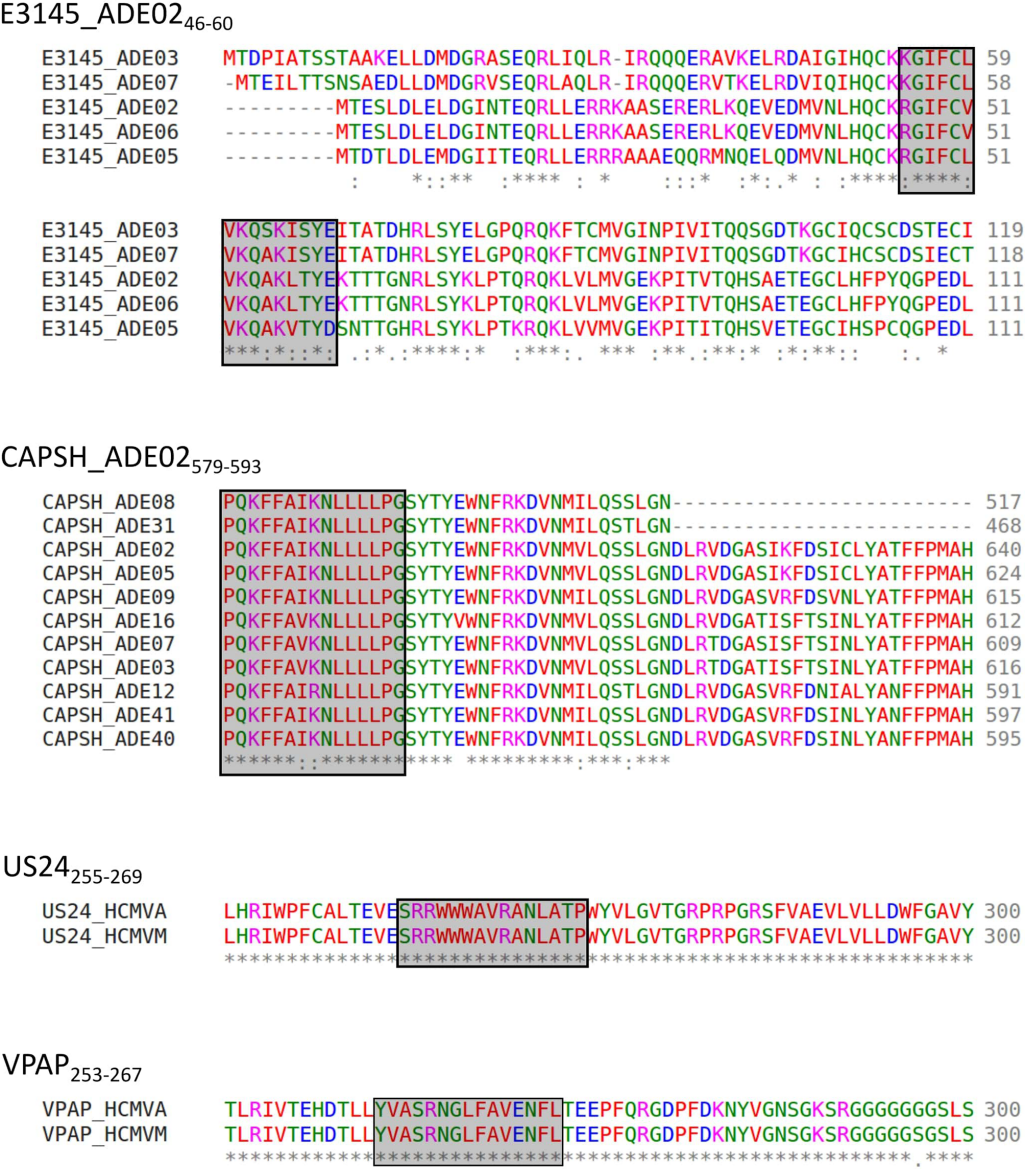
Representative alignment of source protein sequences of HAdV- and HCMV-derived epitopes. Aa sequences of the source proteins of immunodominant epitopes present in the peptide pools were aligned to assess the conservation of epitope sequences among different strains. Representative alignment of four frequently recognized dominant epitopes. * indicates perfect alignment,: indicates strong similarity,. indicates weak similarity, The aa: A, V, F, P, M, I, L, W (“small + hydrophobic (incl. aromatic-Y)”) (45) are shown in red, D, E (acidic aa) in blue, R, K (basic aa) in magenta, S, T, Y, H, C, N, G, Q (“hydroxyl + sulfhydryl + amine + G”) (45) in green, and all other residues (“unusual amino/imino acids, etc.”) (45) are depicted in grey (46).

We performed an alignment of the immunodominant epitopes’ source proteins as available in Swiss-Prot to assess epitope sequence conservation among different serotypes (HAdV) or strains (HCMV). **Figure 8** shows two representative alignments for HAdV and HCMV each.

#### HCMV

The alignment was performed with the corresponding proteins of the HCMV low-passage, wild-type prototype strain Merlin (HCMVM) to explain the detected immune response to HCMV epitopes derived from a laboratory strain. Besides GO_425-439_, all epitopes can be found identically in both strains. GO_425-439_ is altered at positions 6, 13, and 14. US4 is not listed for HCMVM in Swiss-Prot.

#### HAdV

The alignment could be performed for 14 reviewed CAPSH protein sequences, five different PKG1 protein sequences, seven E1BS proteins, seven E3GL proteins, five E3145 proteins, three CAP3 proteins, two COR10 proteins, and two LEAD proteins. **Figure 8** shows the alignment of the protein E3145 of the serotypes 2, 3, 5, 6, and 7. Positions 2 to 5, 7 to 9, 11, and 14 are conserved among all five serotypes. The entire epitope sequence is conserved in the E3145 protein sequence of HAdV6. The HAdV strains 5, 7, and 3 showed 3, 4, and 5 aa polymorphisms, respectively.

The epitope conservation among different serotypes could be shown best for CAPSH_579-593_ (**Figure 8**). By aligning the reviewed hexon protein sequences from the serotypes HAdV2, 3, 5, 7, 8, 9, 12, 16, 31, 40, and 41, we identified the conserved epitope in the hexon of HAdV2, 5, 8, 9, 31, 40, and 41. The epitope sequence in HAdV3, 7, 12, and 16 differ from the HAdV2 sequence in one aa. The reviewed sequences of HAdV1, 4, and 6 were not considered, as the sequences did not or only partly align.

The other epitopes were mostly identical in sequence or showed single aa polymorphisms. The aa of the epitopes are mostly replaced by aa with similar chemical properties to the original aa.

## Discussion

The adoptive T-cell transfer is a promising treatment approach for chemorefractory HCMV and HAdV infection in immunocompromised patients (10–16). However, CD4^+^ T-cell target antigens and epitopes have been scarcely characterized. Here, we contribute a comprehensive set of novel promiscuous HCMV- and HAdV-derived CD4^+^ T-cell epitopes. We show that the CD4^+^ T-cell response is directed against a broad spectrum of different viral antigens of all temporal protein expression classes and various functions. Furthermore, we have designed two peptide pools of virus-specific CD4^+^ T-cell epitopes that can induce an immune response in the vast majority of PBMC cultures from randomly selected blood donors.

### Promiscuous CD4^+^ T-Cell Epitopes Induce Strong IFNγ Responses in PBMCs of Randomly Selected Donors

We used *in silico* prediction and ELISpot screening assays to identify promiscuous CD4^+^ T-cell epitopes.

Although the epitope candidate selection approaches for HCMV and HAdV are slightly different due to the differing viral genome size, we can state that our prediction strategy efficiently detects epitopes that are frequently recognized by T cells. The 12,291 potential 15-mer epitopes that can be derived from the 46 reviewed HAdV2 proteins could be narrowed down to 30 epitope candidates, of which 27 were investigated. For HCMV, 63,901 possible 15-mers from 193 reviewed HCMV AD169 proteins were narrowed down to 169 predicted epitopes, of which 103 were assessed.

Of all tested HCMV-derived epitope candidates (n=103), 74 (71.2%) were immunogenic, of which 17 (16.4%) were dominant. From the tested HAdV epitope candidates (n=27), 21 (77.8%) were immunogenic, and nine (33.3%) were dominant. COR10_53-67_, one of the nine dominant HAdV epitopes, turned out to be an HLA class I epitope as it stimulated only CD8^+^ T-cells in ICS. Single epitopes showed recognition rates of up to 80%, which indicates peptide-binding to different HLA allotypes since the most frequent HLA class II allele (DRB1*15:01) is expressed by around 26% of the population [allelefrequencies.net, Germany pop 8 (30)]. This is supported by the fact that the peptide pools, containing only 11 to 14 peptides, stimulated nearly 100% of all PBMC cultures.

Our approach focused on the identification of frequently recognized promiscuous epitopes from all reviewed viral antigens. Several published epitopes that were retested in this project and confirmed as dominant epitopes were not predicted with our promiscuous prediction. However, four of five dominant epitopes from HCMV were represented with their binding cores in epitope candidates among the top 2% and would have been selected with a lower HLA-promiscuity cut-off (< 5). TEG7_37-51_ was not predicted among the top 2%. For HAdV all published epitopes, except for CAPSH_927-941_, were present among epitope candidates with fewer overlaps (4, 5). Hence, we expect more dominant epitopes among the HCMV epitope candidates predicted to bind to fewer HLA allotypes and the HAdV epitope candidates predicted with fewer overlaps.

There are two other main strategies for the identification of T-cell epitopes. The use of pools of overlapping peptides spanning whole protein sequences (22) affords the synthesis of an immense number of peptides. It is far more time-consuming and costly to use this approach for the identification of promiscuous T-cell epitopes, especially in viruses with large genomes. Lübke et al. came up with a novel approach for T-cell epitope identification. Using viral deletion models that lack immune-evasive features of HCMV, direct identification of naturally presented HCMV-derived HLA ligands was possible (47). However, a prerequisite for this approach is a suitable infection model. So far, this has only been established for HCMV and HLA-class I ligands. Moreover, the cell lines for the direct isolation of HLA ligands only express a maximum of four HLA-DRB1 allotypes. Therefore, it is feasible for the identification of natural ligands bound by the HLA allotypes of the respective cell line but not for the identification of promiscuous T-cell epitopes. The accuracy of reverse immunology approaches can be improved with new data from natural ligand isolation and a thereby better understanding of peptide-binding to HLA class II molecules.

### Dominant T-Cell Epitopes Trigger IFNγ and TNF Secretion Predominantly by CD4^+^ T Cells

We identified the T-cell subsets that responded to the dominant epitopes *via* ICS and distinguished between CD4^+^ and CD8^+^ T-cell responses. Two HCMV epitopes and one HAdV epitope elicited IFNγ and TNF release in both CD8^+^ and CD4^+^ T cells. Provenzano and coworkers have already described the parallel activation of CD4^+^ and CD8^+^ T cells by an HCMV-derived peptide (pp65_340-355_). They assumed the peptide is internalized into immature DCs and trimmed by the immunoproteasome allowing the association to multiple different HLA class I and II allotypes (48). The activation of CD8^+^ T cells in PBMC cultures that were not validated in ICS cannot be excluded. Nonetheless, CD8^+^ T-cell activation was only detected sporadically in ICS, while CD4^+^ T-cell responses were identified in most PBMC cultures. Therefore, we assume that the IFNγ release in ELISpot assays was predominantly elicited by CD4^+^ T cells. From a therapeutic point of view, parallel activation of both T-cell subsets is advantageous as CD4^+^ T cells augment the CD8^+^ T-cell response (11, 12). The responsive T-cell populations we detected in ICS all produced IFNγ and TNF. Multifunctional CD4^+^ T cells were shown to contribute to better HIV control (49) suggesting that the stimulation of multifunctional T cells increases the efficacy of adoptive T-cell therapy.

### Novel Epitopes Were Identified From a Broad Spectrum of Viral Source Antigens

So far, the source antigens for HCMV-derived CD4^+^ T-cell epitopes have been mainly restricted to pp65 (19, 50) and IE-1 (51). We identified T-cell epitopes from a broad spectrum of source antigens, including numerous novel targets. Sylwester et al. discovered CD4^+^ T-cell target antigens translated from 125 different Open Reading Frames of which GB, pp65, MCP, CEP3, pp150, and UL153 were immunodominant (22). Multiple epitopes from these dominant antigens, apart from UL153, were identified in our study. The source antigens cover all temporal stages of HCMV-protein expression as defined by Weekes et al. (34). Further, the source proteins are part of different viral structures and carry out various functions.

In HAdV, the hexon protein has been characterized as the main target of the immune response (21). However, seven newly discovered dominant epitopes that were identified by the promiscuous CD4^+^ T-cell epitope prediction are derived from other proteins than the hexon protein with comparable recognition rates.

It is reasonable to target multiple antigens when designing peptide pools for immunotherapy instead of focussing on well-characterized immunodominant antigens. Lilleri et al. showed that effective protection against an HCMV infection after transplantation depends on T cells targeting multiple antigens and not only pp65 and IE-1 (52).

### The Peptide Sequences of Dominant Epitopes Are Conserved Among HCMV Strains or HAdV Serotypes

We conducted a protein sequence alignment to investigate the conservation of epitope sequences among two HCMV strains and different HAdV serotypes.

HCMV strain AD169 is a standard laboratory strain that underwent several mutations due to the propagation in fibroblasts compared to a wild-type prototype virus (53–55). The source-protein sequences of the identified dominant epitopes were aligned with the correspondent proteins of the HCMV low-passage strain Merlin. All epitopes were entirely conserved among the two strains, except for GO_425-439_. Despite the three single aa polymorphisms in GO_425-439,_ a recognition rate of 62.5% in ELISpot was achieved. Hence, we assume that the single aa polymorphisms, in this case, do not affect peptide binding to the HLA molecule and TCR interaction.

In conclusion, the use of the laboratory strain AD169 is feasible to identify epitopes that also appear in the wild-type virus, which enables epitope identification for clinical purposes.

As we observed high recognition rates of single HAdV epitopes in randomly selected PBMC cultures without prior HAdV serology testing, we performed an alignment of the source proteins of different serotypes in the search for conserved epitopes. The alignment is limited by the differing and generally low numbers of adenoviral source proteins in Swiss-Prot. Nevertheless, it could be shown that epitopes are conserved between different serotypes. We were even able to identify conserved epitope sequences across different HAdV genera. Single aa polymorphisms could also be identified. The influence of single aa polymorphisms on the formation of an HLA-peptide complex and TCR interaction is not yet entirely understood. All epitopes that are present in the HAdV pool are derived from HAdV2. One explanation could be that all PBMC donors had already encountered HAdV species C serotype 2 (HAdV2) since it is highly prevalent in the general population (56). Otherwise, the detected immune response to HAdV2-derived epitopes can be explained by adenovirus-specific T-cell cross-reactivity. This implies that the donor’s encounter with a particular HAdV serotype with identical epitope sequences or single aa polymorphisms can still lead to an immune response against HAdV antigens from another serotype, consistent with previous work (57–60).

### Peptide Pools Activate the Vast Majority of Tested PBMC Cultures

We designed peptide pools comprising dominant T-cell epitopes that were able to stimulate 95.8% (HCMV) and 100% (HAdV) of the tested PBMC cultures of randomly selected blood donors. These responses were induced by only 14 and 12 epitopes, respectively. Besides the therapeutic application, the identified CD4^+^ T-cell epitopes can be used for the monitoring of cell-mediated immunity to personalize antiviral treatment after SOT or SCT (61). The universal peptide pools offer a major advantage in clinical use since they circumvent the time-consuming preparation of individualized, HLA-matched epitope pools to select virus-specific T cells for adoptive T-cell transfer.

## Data Availability Statement

The raw data supporting the conclusions of this article will be made available by the authors, without undue reservation.

## Ethics Statement

The studies involving human participants were reviewed and approved by the Ethik-Kommission an der Medizinischen Fakultät der Eberhard-Karls-Universität und am Universitätsklinikum Tübingen (ProjectNo.507/2017B01). The patients/participants provided their written informed consent to participate in this study.

## Author Contributions

AH performed the experiments, analyzed and interpreted the data related to HCMV. LM performed the experiments, analyzed and interpreted the data related to HAdV. AH and LM wrote the manuscript. SS designed and supervised the study. SS and ML helped interpreting data, assisted in preparing the manuscript and reviewed the manuscript. H-GR supervised the study and reviewed the manuscript. All authors contributed to the article and approved the submitted version.

## Funding

AH was supported by the IZKF Promotionskolleg of the Faculty of Medicine Tübingen (2018-2). LM was supported by the Integrated Research Training Group SFB 685. Part of this work was supported by the National Center for Infection Research (DZIF), partner site Tübingen. We acknowledge support by the Open Access Publishing Fund of the University of Tübingen.

## Conflict of Interest

The authors declare that the research was conducted in the absence of any commercial or financial relationships that could be construed as a potential conflict of interest.
