# Supplementary material for: Broad and Efficient Activation of Memory CD4+ T Cells by Novel HAdV- and HCMV-Derived Peptide Pools
Source: Front Immunol. 2021 Jul 7;12:700438. doi: 10.3389/fimmu.2021.700438 (PMC8312486; doi:10.3389/fimmu.2021.700438)
Supplement: Supplementary file 1 [file Table_1.docx]

**A: Positive control peptide for HCMV ELISpots.**

| Sequence | Source protein | Position |
| --- | --- | --- |
| YLSPGLVQFA | CAPSH_ADE02 | 23-32 |

B: **Positive HLA class II pool for HCMV ELISpots**. This peptide pool consists of peptides that were frequently recognized in ELISpot assays. For each peptide, activation of CD4^+^ T cells was confirmed by ICS.

| Sequence | Source protein | Position |
| --- | --- | --- |
| YQEFFWDANDIYRIF | pp65_HCMVA | 510-524 |
| PRPVSRFLGNNSILY | GP350_EBVB9 | 268-282 |
| IAEGLRALLARSHVERTTDE | EBN1_EBV | 481-500 |
| RRGTALAIPQCRLTPLSRLP | EBN1_EBV | 521-540 |
| RSPTVFYNIPPMPLPPSQL | EBNA2_EBV | 277-295 |
| TLLYLKYKSRRSFID | E3GL_ADE06 | 140-154 |
| RQVMDRIMSLTARNP | CAP3_ADE02 | 28-42 |
| MHLWRAVVRHKNRLL | E1BS_ADE02 | 120-134 |
| KNRLLLLSSVRPAII | E1BS_ADE02 | 130-144 |
| TLVLAFVKTCAVLAA | LEAD_ADE02 | 32-46 |
| RGIFCVVKQAKLTYE | E3145_ADE02 | 46-60 |
| VSKFFHAFPSKLHDK | PKG1_ADE02 | 292-306 |
| TFYLNHTFKKVAITF | CAPSH_ADE02 | 727-741 |
| PQKFFAIKNLLLLPG | CAPSH_ADE05 | 563-577 |

C: **Positive HLA class II peptides for HAdV ELISpots**. These peptides were frequently recognized in ELISpot assays. For each peptide, activation of CD4^+^ T cells was confirmed by ICS.

| Sequence | Source protein | Position |
| --- | --- | --- |
| GTAYNALAPKGAPNP | CAPSH_ADE05 | 117-131 |
| TFYLNHTFKK | CAPSH_ADE02 | 726-735 |
| RSPTVFYNIPPMPLPPSQL | EBNA2_EBVB9 | 277-295 |
| PRPVSRFLGNNSILY | GP350_EBVB9 | 268-282 |
| IAEGLRALLARSHVERTTDE | EBNA1_EBVB9 | 481-500 |
