# Supplementary material for: Broad and Efficient Activation of Memory CD4+ T Cells by Novel HAdV- and HCMV-Derived Peptide Pools
Source: Front Immunol. 2021 Jul 7;12:700438. doi: 10.3389/fimmu.2021.700438 (PMC8312486; doi:10.3389/fimmu.2021.700438)
Supplement: Supplementary file 2 [file Table_2.docx]

**Table S2: Epitopes from the HCMV prediction**

All epitopes that were predicted binders to five or six HLA allotypes are listed here. Abbreviations: n_HLA_ – number of covered HLA-DR allotypes (six are highlighted in yellow), n_ts_ – number of top-scored epitope candidates (one and two are highlighted in yellow), HLA_nc_ – not covered HLA allotypes, ns – not synthesized, sf – synthesis failed.

| **Protein** | **Position** | **Sequence** | **n_HLA_** | **n_ts_** | **HLA_nc_** | **Status** |
| --- | --- | --- | --- | --- | --- | --- |
| **EP84** | 347 | ALLLPIERGAVVSSP | 6 | 0 | - |  |
| **EP84** | 298 | MSLPLDTSEAVAFLN | 5 | 0 | DR11 |  |
| **EP84** | 471 | LCDLPLVSSRLLPET | 5 | 0 | DR11 |  |
| **VPAP** | 249 | DTLLYVASRNGLFAV | 5 | 0 | DR1 |  |
| **US9** | 196 | YVVLVQFVKHVALFS | 5 | 1 | DR7 |  |
| **GH** | 8 | YLTVFTVYLLSHLPS | 5 | 1 | DR3 |  |
| **LTP** | 658 | VLRLFYDLRDLKLCD | 5 | 0 | DR11 |  |
| **LTP** | 830 | NAVLSMFHTLVMRLA | 5 | 1 | DR3 |  |
| **HHLF1** | 214 | FRVFVYDLANNTLIL | 5 | 1 | DR1 |  |
| **HHLF1** | 306 | MVLLGAWQELAQYEP | 5 | 0 | DR7 |  |
| **UL7** | 23 | YNKLLILALFTPVIL | 5 | 1 | DR11 |  |
| **UL8** | 23 | YNKLLILALFTPVIL | 5 | 1 | DR11 |  |
| **PRIM** | 198 | MGEFARLLLGSPFRQ | 5 | 0 | DR7 | sf |
| **PRIM** | 254 | VHYVYLAYRTALARA | 5 | 0 | DR7 | sf |
| **PRIM** | 378 | ESVFSPLERSLSGLL | 6 | 1 | - | sf |
| **PRIM** | 893 | VQVFIDLRTEHSYAL | 6 | 1 | - | sf |
| **US8** | 191 | MVLLLGYVLARTVYR | 5 | 0 | DR11 |  |
| **IE1** | 313 | CCYVLEETSVMLAKR | 5 | 1 | DR11 |  |
| **IE2** | 501 | VDLLGALNLCLPLMQ | 5 | 0 | DR11 |  |
| **UL97** | 82 | VTTLTTLSSVSTTTV | 5 | 0 | DR3 |  |
| **UL97** | 559 | VLGFCLMRLLDRRGL | 5 | 0 | DR7 |  |
| **TRM3** | 222 | IIPIISFLLKHMIGI | 5 | 0 | DR3 |  |
| **TRM3** | 319 | FNTILGFLAQNTTKI | 5 | 0 | DR3 |  |
| **UL119** | 252 | LREFVFYLNGTYTVV | 5 | 0 | DR11 | sf |
| **AN** | 238 | LGLLIDPTSGLLGAS | 5 | 1 | DR11 |  |
| **UL298** | 430 | MLGDTQYFGVVRDHK | 5 | 1 | DR1 |  |
| **DPOL** | 67 | LMFYREIKHLLSHDM | 5 | 1 | DR3 |  |
| **DPOL** | 988 | CEFVKGVTRDVLSLL | 5 | 0 | DR15 |  |
| **DPOL** | 1056 | LVLSSVLSKDISLYR | 5 | 0 | DR3 |  |
| **UL11** | 133 | CYYVYVTQNGTLPTT | 6 | 2 | - |  |
| **UL117** | 330 | VATFKFFHQDPNRVL | 5 | 1 | DR11 |  |
| **GO** | 425 | LLFLDEIRNFSLRSP | 5 | 1 | DR1 |  |
| **UL84** | 122 | RDPFQILLSTPLQLG | 5 | 1 | DR11 |  |
| **UL84** | 474 | WLELTVLVSDENGAT | 5 | 0 | DR15 | sf |
| **DNBI** | 689 | RSVFYVIQNVALITA | 6 | 1 | - |  |
| **DNBI** | 844 | LQFWQKVCSNALPKN | 5 | 1 | DR3 |  |
| **DNBI** | 871 | VKFLVAVTADYQEHD | 5 | 0 | DR4 |  |
| **DNBI** | 1115 | ASLMDKFAALQEQGV | 5 | 0 | DR4 |  |
| **CVC2** | 400 | RDDVLSLWSRRLLVG | 5 | 0 | DR11 |  |
| **CVC2** | 595 | EHGLGRLLSVTLPRH | 5 | 0 | DR3 |  |
| **HEPA** | 23 | LSWYGLLEASVPIVQ | 5 | 0 | DR3 | sf |
| **HEPA** | 150 | LSLFHVAKLVVIGSY | 5 | 0 | DR3 |  |
| **HEPA** | 264 | WTHLYDVLFRGFAGQ | 5 | 1 | DR3 |  |
| **HEPA** | 438 | VLIVDLVERVLAKCV | 5 | 0 | DR15 |  |
| **HEPA** | 488 | FWQIQSLLGYISEHV | 5 | 0 | DR3 |  |
| **HEPA** | 706 | YREILFRFVARRNDV | 5 | 0 | DR3 |  |
| **UL20** | 190 | FMDYVILTPLAVLTC | 5 | 0 | DR11 |  |
| **HHLF1** | 170 | AWIVLVATVVHEVDP | 5 | 0 | DR3 | ns |
| **UL52** | 240 | LIIMSEFTHLLQQHF | 5 | 0 | DR11 |  |
| **RIR1** | 341 | IYRFHLDARFEGEVL | 5 | 0 | DR1 |  |
| **RIR1** | 382 | VPQYDFLISADPFSR | 5 | 0 | DR3 |  |
| **RIR1** | 400 | WAAMCKWMSTLSCGV | 5 | 1 | DR3 |  |
| **RIR1** | 454 | FVDMWDVAAIRVINF | 5 | 0 | DR3 | ns |
| **RIR1** | 658 | WWVESALEKLRPLHI | 5 | 0 | DR3 | ns |
| **RIR1** | 689 | FASWDLIERIFEHMY | 5 | 0 | DR1 | ns |
| **NEC2** | 103 | FNVLKVNESLIVTLK | 6 | 0 | - |  |
| **HELI** | 167 | IYRVFGFVSKHVPLA | 5 | 0 | DR3 | ns |
| **HELI** | 877 | RTAMTIAKSQGLSLE | 5 | 0 | DR11 | ns |
| **DUT** | 311 | RFTYLPVGSHPLGQM | 5 | 1 | DR3 |  |
| **NEC1** | 175 | FQIYYLLHAANHDIV | 5 | 0 | DR3 | ns |
| **TRX1** | 141 | GITSLLTCVMRGYLY | 5 | 0 | DR15 | ns |
| **UL8** | 86 | STPYVGLSLSCAANQ | 5 | 1 | DR3 |  |
| **UL8** | 231 | SSDWVTLGTSASLLR | 6 | 2 | - |  |
| **MCP** | 515 | DFVVTDFYKVGNITL | 5 | 1 | DR11 |  |
| **MCP** | 570 | FHELRTWEIMEHMRL | 5 | 1 | DR3 |  |
| **MCP** | 621 | VDAFLLIRTFVARCI | 5 | 1 | DR3 |  |
| **MCP** | 1321 | ALPILSTTTLALMET | 5 | 0 | DR11 | ns |
| **PORTL** | 246 | VRVFKKVRSERLEAQ | 5 | 1 | DR3 |  |
| **UL15A** | 85 | MFLVFGLCSWLAMRY | 6 | 0 | - |  |
| **PP85** | 352 | TEVYQTLRDYNVLFY | 5 | 0 | DR11 | ns |
| **CVC1** | 123 | RMFYAVFTTLGLRCP | 5 | 1 | DR3 |  |
| **UL95** | 284 | HVEAVLRQVYTPGLL | 5 | 0 | DR15 | ns |
| **UL132** | 83 | AILFYIVTGTSIFSF | 5 | 0 | DR3 | ns |
| **UL9** | 7 | LLWWITILLRIQQFY | 5 | 1 | DR1 |  |
| **UL9** | 105 | YSGIYYFDSLYTYGW | 5 | 1 | DR3 |  |
| **UL9** | 111 | FDSLYTYGWVLRTPL | 5 | 1 | DR3 |  |
| **US29** | 363 | LRHATSLVTVPTLLL | 5 | 0 | DR11 | ns |
| **UL34** | 219 | NSFLHLLMNSGLDIA | 6 | 0 | - |  |
| **UL34** | 248 | LFQIGHTDSVSAALE | 5 | 0 | DR3 | ns |
| **US22** | 292 | LVLLDKFGVVYLHKI | 5 | 0 | DR7 | ns |
| **US22** | 360 | LRWHGALGTITRSQL | 5 | 0 | DR1 | ns |
| **US17** | 113 | LTIYSVLTTLSVIVA | 5 | 1 | DR3 |  |
| **US15** | 191 | FKIVLSFSVLITCLA | 5 | 2 | DR3 |  |
| **UL27** | 227 | FLEPEERELIGRCLP | 5 | 0 | DR15 | ns |
| **UL27** | 423 | VQRLIRLFKGEAALL | 5 | 0 | DR3 | ns |
| **UL116** | 117 | VSILTTVTPAATSTI | 5 | 0 | DR3 | ns |
| **TR14** | 99 | LIGDTTLSTLGTCPV | 5 | 0 | DR11 | ns |
| **UL42** | 100 | FLAVVFTVVINRDSA | 5 | 1 | DR3 |  |
| **VP22** | 201 | KRYFRPLLRAWSLGL | 5 | 1 | DR3 |  |
| **US24** | 255 | SRRWWWAVRANLATP | 5 | 1 | DR3 |  |
| **US19** | 192 | TLMLIHDLSLITCQS | 5 | 0 | DR1 | ns |
| **UL96** | 3 | SVNKQLLKDVMRVDL | 5 | 0 | DR7 | ns |
| **UL88** | 406 | LGYDRLVSADAGVSR | 5 | 1 | DR3 |  |
| **UL36** | 42 | ERCFIQLRSRSALGP | 5 | 1 | DR7 |  |
| **UL36** | 253 | QYVLVDTFGVVYGYD | 6 | 1 | - |  |
| **US31** | 103 | FTWWKRLRHSTRRWL | 5 | 0 | DR1 | ns |
| **US34** | 39 | FYGYLQLDLLGPVVA | 5 | 0 | DR7 | ns |
| **UL87** | 190 | MACLPRDLSLHLDDY | 5 | 0 | DR1 | ns |
| **UL87** | 458 | VRRYVCIISRLMYAR | 5 | 1 | DR11 | sf |
| **UL87** | 760 | AKQLVLFLRACLLKL | 5 | 0 | DR11 | ns |
| **UL19** | 4 | NALYELFRRRLPRAP | 5 | 0 | DR7 | ns |
| **UL14** | 138 | YTCVLGNETHSLATE | 5 | 0 | DR15 | ns |
| **UL14** | 265 | KIGLLAAGSVALTSL | 5 | 0 | DR11 | ns |
| **UL28** | 108 | MLGDTQYFGVVRDHK | 5 | 1 | DR1 |  |
| **UL28** | 279 | FVVIGWMEPVNKAVF | 5 | 0 | DR4 | ns |
| **UL49** | 197 | RFLFGVDLRLPVLHP | 5 | 1 | DR1 |  |
| **UL40** | 185 | MYTVGILALGSFSSF | 5 | 0 | DR7 | ns |
| **UL40** | 196 | FSSFYSQIARSLGVL | 5 | 1 | DR3 |  |
| **UL31** | 202 | CGYKYDWSNVVTPKA*^2^ | 5 | 1 | DR3 |  |
| **US26** | 33 | IRHLVRSYADMNISL | 5 | 0 | DR7 | ns |
| **UL78** | 272 | IMDYVELATRTLLTM | 5 | 1 | DR11 |  |
| **J1I** | 240 | RLLFAVRAARRFYSP | 5 | 0 | DR7 | ns |
| **YHR1** | 50 | VLRFFTVVRDVDLPR | 6 | 1 | - |  |
| **UL107** | 27 | QNSFFSFLSRKKSMY | 5 | 2 | DR7 |  |
| **UL108** | 46 | SSFFDVLLSSRSCFV | 5 | 1 | DR15 |  |
| **US33** | 33 | FDVVLTFVPSGFVMG | 6 | 0 | - |  |
| **UL101** | 88 | LGAYRTMSVFGSGWR | 5 | 1 | DR3 |  |
| **UL110** | 91 | IMMIIIIHSPTIFIL | 6 | 0 | - |  |
| **US4** | 62 | PYFPMRFINVKSHVS | 5 | 1 | DR3 |  |
| **US25** | 50 | LRTDLAFGPVRRNSR | 5 | 0 | DR7 | ns |
| **UL39** | 40 | HRSIFFILSVMIGKG | 5 | 0 | DR3 | ns |
| **US5** | 21 | TGVVYRDISSTIATE | 5 | 1 | DR15 |  |
| **US36** | 46 | RTALNLFLSMSLCVP | 5 | 1 | DR1 |  |
| **UL81** | 47 | LPAPHAVDPASRERL | 5 | 0 | DR7 | ns |
| **UL81** | 73 | LYLFLNNKNTETLII | 5 | 1 | DR1 |  |
| **UL90** | 23 | VSADWFRFSGRSPVG | 5 | 0 | DR11 | ns |
| **UL60** | 4 | VEKYWRMRTTHTVEF | 5 | 0 | DR11 | ns |
| **UL61** | 69 | VRAEFFWGAAGEGSV | 5 | 0 | DR3 | ns |
| **UL61** | 138 | TPLPELLTGPPAPNL | 5 | 0 | DR7 | ns |
| **UL61** | 384 | GFIGFQMPRLGGRSG | 6 | 0 | - |  |
| **UL65** | 64 | SIVLYEHLDARVTDD | 5 | 0 | DR3 | ns |
| **UL66** | 55 | MMWFVVLTVSFSYYR | 5 | 0 | DR4 | ns |
| **UL67** | 8 | IIYIIYSDDSVVNIS | 5 | 0 | DR11 | ns |
| **UL67** | 76 | FVYLHSVESYSLQFH | 5 | 1 | DR7 |  |
| **IR04** | 57 | FIIFFYFLSSPFLNL | 5 | 1 | DR11 |  |
| **IR04** | 63 | FLSSPFLNLGLSFPS | 5 | 0 | DR4 | ns |
| **IR12** | 17 | AYTIIIFYILHRVTC | 5 | 0 | DR3 | ns |
| **IR12** | 92 | TTVYSTFNTSYANIS | 5 | 1 | DR11 |  |
| **IR12** | 99 | NTSYANISNTAATTE | 5 | 0 | DR3 | ns |
| **IR02** | 15 | IASFAATLLHRYPIN | 5 | 0 | DR1 | ns |
| **IR09** | 103 | YRQGPGFLLEKQHVG | 5 | 0 | DR15 | ns |
| **IR14** | 93 | RWPVDRFLRVPLQRA | 5 | 0 | DR7 | ns |
| **IR07** | 48 | IFTQSFLRTFRNQQV | 5 | 0 | DR1 | ns |
| **IR13** | 5 | FTVMWTILISALSES | 5 | 1 | DR15 |  |
| **YHL4** | 111 | WLLVLNLNVALPVTA | 6 | 1 | - |  |
| **PP65** | 347 | ALFFFDIDLLLQRGP | 6 | 0 | - |  |
| **PP71** | 204 | AIPLTLVDALEQLAC | 5 | 0 | DR1 | ns |
| **PP71** | 488 | WHVFASLDDLVPLTV | 5 | 0 | DR3 | ns |
| **PP150** | 459 | GGVSSIFSGLLSSGS | 5 | 1 | DR3 |  |
| **IRS1** | 214 | FRVFVYDLANNTLIL | 5 | 1 | DR1 |  |
| **US6** | 152 | LCCGITLLVVILALL | 5 | 0 | DR1 | ns |
| **ICP27** | 512 | YRFMIAYCPFDEQSL | 5 | 0 | DR3 | ns |
| **SCAF** | 376 | KDAFFSLLGASRSAV | 5 | 0 | DR3 | ns |
| **UL35** | 181 | YPRLTTYNLLFHPPP | 5 | 1 | DR3 |  |
| **US6** | 155 | GITLLVVILALLCSI | 5 | 0 | DR7 | sf |
| **US6** | 134 | WNAFRLIERHGFFAV | 5 | 0 | DR7 |  |
| **GB** | 331 | VISWDIQDEKNVTCQ | 5 | 0 | DR11 |  |
| **GM** | 57 | MSAYNVMHLHTPMLF | 5 | 0 | DR3 |  |
| **IRS1** | 168 | RDAWIVLVATVVHEV | 5 | 0 | DR3 |  |
| **VGLI** | 3 | PVYVNLLGSVGLLAF | 5 | 0 | DR3 |  |
| **UL22A** | 7 | ILSLLAVTLTVALAA | 5 | 0 | DR4 |  |
| **PP150** | 59 | WLGYYRELRFHNPDL | 5 | 1 | DR7 |  |
| **UL35** | 108 | QLDVLYSDPLKTRLL | 6 | 0 | - |  |
| **UL35** | 439 | TYHLQRIYSMMIEGA | 5 | 0 | DR3 |  |
| **UL35** | 462 | KRFMELLDRAPLGQE | 5 | 0 | DR7 |  |
| **SCAF** | 118 | DKVVEFLSGSYAGLS | 5 | 0 | DR4 |  |
| **PP71** | 282 | GFQLLIPKSFTLTRI | 5 | 0 | DR11 |  |
| **CEP3** | 18 | GEPLKDALGRQVSLR | 5 | 0 | DR3 |  |
| **CEP3** | 31 | LRSYDNIPPTSSSDE | 6 | 1 | - |  |
| **US2** | 178 | LFIVYVTVDCNLSMM | 5 | 0 | DR1 | sf |
| **US3** | 152 | DDNWGLLFRTLLVYL | 5 | 1 | DR3 |  |
| **UL31** | 203 | GYKYDWSNVVTPKAA | 5 | 1 | DR3 |  |
