# Supplementary material for: Broad and Efficient Activation of Memory CD4+ T Cells by Novel HAdV- and HCMV-Derived Peptide Pools
Source: Front Immunol. 2021 Jul 7;12:700438. doi: 10.3389/fimmu.2021.700438 (PMC8312486; doi:10.3389/fimmu.2021.700438)
Supplement: Supplementary file 3 [file Table_3.docx]

**Table S3: ELISpot and ICS results of HCMV epitope candidates**

Predicted epitopes are listed with ELISpot recognition rate and ICS results. Epitopes with ELISpot recognition rates ≥ 50 % are shown in Table 2. Abbreviations: n_pd_ – number of positively tested PBMC cultures, n_td_ – number of tested PBMC cultures, rr – ELISpot recognition rate.

| Protein_Position_ | Sequence | ELISpot | | ICS | Reference |
| --- | --- | --- | --- | --- | --- |
|  |  | n_pd_/n_td_ | rr |  |  |
| UL52_240-254_ | LIIMSEFTHLLQQHF | 15/31 | 48.4 | nt | this paper |
| IE1_313-327_ | CCYVLEETSVMLAKR | 11/23 | 47.8 | CD4 | (1) (313-327) |
| NEC2_103-117_ | FNVLKVNESLIVTLK | 11/23 | 47.8 | nt | this paper |
| YHR1_50-64_ | VLRFFTVVRDVDLPR | 10/21 | 47.6 | CD4 | this paper |
| UL61_384-398_ | GFIGFQMPRLGGRSG | 10/22 | 45.5 | CD4 | this paper |
| UL49_197-211_ | RFLFGVDLRLPVLHP | 10/22 | 45.5 | CD4 | this paper |
| EP84_347-361_ | ALLLPIERGAVVSSP | 10/22 | 45.5 | CD4 | this paper |
| MCP_621-635_ | VDAFLLIRTFVARCI | 9/20 | 45.0 | nt | this paper |
| UL31_203-217_ | GYKYDWSNVVTPKAA | 10/23 | 43.5 | CD4 | this paper |
| DUT_311-325_ | RFTYLPVGSHPLGQM | 9/21 | 42.9 | nt | this paper |
| UL40_196-210_ | FSSFYSQIARSLGVL | 6/14 | 42.9 | nt | this paper |
| UL78_272-286_ | IMDYVELATRTLLTM | 5/13 | 38.5 | nt | this paper |
| HEPA_706-720_ | YREILFRFVARRNDV | 5/14 | 35.7 | nt | this paper |
| DPOL_988-1002_ | CEFVKGVTRDVLSLL | 8/24 | 33.3 | nt | this paper |
| UL20_190-204_ | FMDYVILTPLAVLTC | 5/15 | 33.3 | nt | this paper |
| UL81_73-87_ | LYLFLNNKNTETLII | 7/21 | 33.3 | CD4 | this paper |
| RIR1_341-355_ | IYRFHLDARFEGEVL | 7/22 | 31.8 | nt | this paper |
| DNBI_871-885_ | VKFLVAVTADYQEHD | 5/16 | 31.3 | nt | this paper |
| UL11_133-147_ | CYYVYVTQNGTLPTT | 5/16 | 31.3 | nt | this paper |
| AN_238-252_ | LGLLIDPTSGLLGAS | 5/16 | 31.3 | nt | this paper |
| UL117_330-344_ | VATFKFFHQDPNRVL | 5/16 | 31.3 | nt | this paper |
| HEPA_438-452_ | VLIVDLVERVLAKCV | 5/17 | 29.4 | CD4 | this paper |
| HEPA_150-164_ | LSLFHVAKLVVIGSY | 4/14 | 28.6 | nt | this paper |
| UL107_27-41_ | QNSFFSFLSRKKSMY | 2/7 | 28.6 | nt | this paper |
| UL108_46-60_ | SSFFDVLLSSRSCFV | 2/7 | 28.6 | nt | this paper |
| US33_33-47_ | FDVVLTFVPSGFVMG | 2/7 | 28.6 | nt | this paper |
| PP65_347-361_ | ALFFFDIDLLLQRGP | 2/7 | 28.6 | nt | (2) (350-358)&(348-356); (3) (340-355); (4) (347-365) |
| PP150_459-473_ | GGVSSIFSGLLSSGS | 2/7 | 28.6 | nt | this paper |
| UL35_181-195_ | YPRLTTYNLLFHPPP | 2/7 | 28.6 | nt | this paper |
| TRM3_222-236_ | IIPIISFLLKHMIGI | 2/7 | 28.6 | nt | this paper |
| UL37_3-17_ | PVYVNLLGSVGLLAF | 3/11 | 27.3 | nt | this paper |
| CEP3_18-32_ | GEPLKDALGRQVSLR | 4/15 | 26.7 | nt | this paper |
| DNBI_844-858_ | LQFWQKVCSNALPKN | 4/16 | 25.0 | nt | this paper |
| HHLF1_306-320_ | MVLLGAWQELAQYEP | 4/16 | 25.0 | nt | this paper |
| US15_191-205_ | FKIVLSFSVLITCLA | 2/8 | 25.0 | nt | this paper |
| CVC1_123-137_ | RMFYAVFTTLGLRCP | 3/14 | 21.4 | nt | this paper |
| IRS1_168-182_ | RDAWIVLVATVVHEV | 4/19 | 21.1 | nt | this paper |
| UL22A_7-21_ | ILSLLAVTLTVALAA | 5/15 | 20.0 | nt | this paper |
| UL9_111-125_ | FDSLYTYGWVLRTPL | 5/15 | 20.0 | nt | this paper |
| UL36_42-56_ | ERCFIQLRSRSALGP | 1/6 | 16.7 | nt | this paper |
| UL67_76-90_ | FVYLHSVESYSLQFH | 1/7 | 14.3 | nt | this paper |
| IR12_92-106_ | TTVYSTFNTSYANIS | 1/7 | 14.3 | nt | this paper |
| YHL4_111-125_ | WLLVLNLNVALPVTA | 1/7 | 14.3 | nt | this paper |
| LTP_658-672_ | VLRLFYDLRDLKLCD | 2/14 | 14.3 | nt | this paper |
| US36_46-60_ | RTALNLFLSMSLCVP | 2/14 | 14.3 | nt | this paper |
| UL88_406-420_ | LGYDRLVSADAGVSR | 2/14 | 14.3 | nt | this paper |
| UL110_91-105_ | IMMIIIIHSPTIFIL | 1/7 | 14.3 | nt | this paper |
| CEP3_31-45_ | LRSYDNIPPTSSSDE | 2/15 | 13.3 | nt | this paper |
| IE2_501-515_ | VDLLGALNLCLPLMQ | 1/8 | 12.5 | nt | this paper |
| UL29/28_430-444_ | MLGDTQYFGVVRDHK | 1/8 | 12.5 | nt | this paper |
| DNBI_1115-1129_ | ASLMDKFAALQEQGV | 1/8 | 12.5 | nt | this paper |
| UL8_231-245_ | SSDWVTLGTSASLLR | 1/8 | 12.5 | nt | this paper |
| UL8_86-100_ | STPYVGLSLSCAANQ | 1/8 | 12.5 | nt | this paper |
| UL9_105-119_ | YSGIYYFDSLYTYGW | 1/8 | 12.5 | nt | this paper |
| RIR1_400-414_ | WAAMCKWMSTLSCGV | 1/8 | 12.5 | nt | this paper |
| UL9_7-21_ | LLWWITILLRIQQFY | 1/13 | 7.7 | nt | this paper |
| US3_152-166_ | DDNWGLLFRTLLVYL | 1/16 | 6.3 | nt | this paper |
| GB_331-345_ | VISWDIQDEKNVTCQ | 0/11 | 0.0 | nt | this paper |
| U7_23-37_ | YNKLLILALFTPVIL | 0/8 | 0.0 | nt | this paper |
| UL97_82-96_ | VTTLTTLSSVSTTTV | 0/8 | 0.0 | nt | this paper |
| UL97_559-573_ | VLGFCLMRLLDRRGL | 0/8 | 0.0 | nt | this paper |
| DPOL_1056-1070_ | LVLSSVLSKDISLYR | 0/8 | 0.0 | nt | this paper |
| DNBI_689-703_ | RSVFYVIQNVALITA | 0/8 | 0.0 | nt | this paper |
| UL15A_85-99_ | MFLVFGLCSWLAMRY | 0/8 | 0.0 | nt | this paper |
| LTP_830-844_ | NAVLSMFHTLVMRLA | 0/7 | 0.0 | nt | this paper |
| PP150_59-73_ | WLGYYRELRFHNPDL | 0/7 | 0.0 | nt | this paper |
| UL35_108-122_ | QLDVLYSDPLKTRLL | 0/7 | 0.0 | nt | this paper |
| UL35_462-476_ | KRFMELLDRAPLGQE | 0/7 | 0.0 | nt | this paper |
| EP84_471-485_ | LCDLPLVSSRLLPET | 0/7 | 0.0 | nt | this paper |
| US9_196-210_ | YVVLVQFVKHVALFS | 0/7 | 0.0 | nt | this paper |
| GH_108-122_ | YLTVFTVYLLSHLPS | 0/7 | 0.0 | nt | (5) (15-60) |
| SCAF_118-132_ | DKVVEFLSGSYAGLS | 0/7 | 0.0 | nt | this paper |
| PP71_282-296_ | GFQLLIPKSFTLTRI | 0/7 | 0.0 | nt | this paper |
| RIR1_382-396_ | VPQYDFLISADPFSR | 0/7 | 0.0 | nt | this paper |
| VPAP_249-263_ | DTLLYVASRNGLFAV | 0/6 | 0.0 | nt | this paper |
| EP84_298-312_ | MSLPLDTSEAVAFLN | 0/6 | 0.0 | nt | this paper |
| UL35_439-453_ | TYHLQRIYSMMIEGA | 0/6 | 0.0 | nt | this paper |
| HEPA_264-278_ | WTHLYDVLFRGFAGQ | 0/6 | 0.0 | nt | this paper |
| US6_134-148_ | WNAFRLIERHGFFAV | 0/5 | 0.0 | nt | this paper |
| GM_57-71_ | MSAYNVMHLHTPMLF | 0/5 | 0.0 | nt | this paper |
| UL101_88-102_ | LGAYRTMSVFGSGWR | 0/7 | 0.0 | nt | this paper |
| US17_113-127_ | LTIYSVLTTLSVIVA | 0/8 | 0.0 | nt | this paper |
| UL42_100-114_ | FLAVVFTVVINRDSA | 0/8 | 0.0 | nt | this paper |
| US5_21-35_ | TGVVYRDISSTIATE | 0/6 | 0.0 | nt | this paper |
| IR04_57-71_ | FIIFFYFLSSPFLNL | 0/7 | 0.0 | nt | this paper |
| IR13_5-19_ | FTVMWTILISALSES | 0/7 | 0.0 | nt | this paper |

1. Malik A, Adland E, Laker L, Kloverpris H, Fardoos R, Roider J, et al. Immunodominant cytomegalovirus-specific CD8+ T-cell responses in sub-Saharan African populations. *PLoS One* (2017) 12(12):e0189612. doi: 10.1371/journal.pone.0189612.

2. Bronke C, Palmer NM, Westerlaken GH, Toebes M, van Schijndel GM, Purwaha V, et al. Direct ex vivo detection of HLA-DR3-restricted cytomegalovirus- and Mycobacterium tuberculosis-specific CD4+ T cells. *Hum Immunol* (2005) 66(9):950-61. doi: 10.1016/j.humimm.2005.06.011.

3. Provenzano M, Sais G, Bracci L, Egli A, Anselmi M, Viehl CT, et al. A HCMV pp65 polypeptide promotes the expansion of CD4+ and CD8+ T cells across a wide range of HLA specificities. *J Cell Mol Med* (2009) 13(8B):2131-47. doi: 10.1111/j.1582-4934.2008.00531.x.

4. Hanley PJ, Cruz CRY, Savoldo B, Leen AM, Stanojevic M, Khalil M, et al. Functionally active virus-specific T cells that target CMV, adenovirus, and EBV can be expanded from naive T-cell populations in cord blood and will target a range of viral epitopes. *Blood* (2009) 114(9):1958-67. doi: 10.1182/blood-2009-03-213256.

5. Beninga J, Kalbacher H, Mach M. Analysis of T helper cell response to glycoprotein H (gpUL75) of human cytomegalovirus: evidence for strain-specific T cell determinants. *J Infect Dis* (1996) 173(5):1051-61. doi: 10.1093/infdis/173.5.1051.
